# Forgetting ourselves in flow: an active inference account of flow states and how we experience ourselves within them

**DOI:** 10.3389/fpsyg.2024.1354719

**Published:** 2024-06-03

**Authors:** Darius Parvizi-Wayne, Lars Sandved-Smith, Riddhi J. Pitliya, Jakub Limanowski, Miles R. A. Tufft, Karl J. Friston

**Affiliations:** ^1^Department of Experimental Psychology, University College London, London, United Kingdom; ^2^Monash Centre for Consciousness and Contemplative Studies, Monash University, Clayton, VIC, Australia; ^3^Department of Experimental Psychology, University of Oxford, Oxford, United Kingdom; ^4^VERSES AI Research Lab, Los Angeles, CA, United States; ^5^Institute of Psychology, University of Greifswald, Greifswald, Germany; ^6^Queen Square Institute of Neurology, University College London, London, United Kingdom

**Keywords:** active inference, embodied cognition, phenomenology, consciousness, self-awareness, predictive processing

## Abstract

Flow has been described as a state of optimal performance, experienced universally across a broad range of domains: from art to athletics, gaming to writing. However, its phenomenal characteristics can, at first glance, be puzzling. Firstly, individuals in flow supposedly report a loss of self-awareness, even though they perform in a manner which seems to evince their agency and skill. Secondly, flow states are felt to be effortless, despite the prerequisite complexity of the tasks that engender them. In this paper, we unpick these features of flow, as well as others, through the active inference framework, which posits that action and perception are forms of active Bayesian inference directed at sustained self-organisation; i.e., the minimisation of variational free energy. We propose that the phenomenology of flow is rooted in the deployment of high precision weight over (i) the expected sensory consequences of action and (ii) beliefs about how action will sequentially unfold. This computational mechanism thus draws the embodied cognitive system to minimise the ensuing (i.e., expected) free energy through the exploitation of the pragmatic affordances at hand. Furthermore, given the challenging dynamics the flow-inducing situation presents, attention must be wholly focussed on the unfolding task whilst counterfactual planning is restricted, leading to the attested loss of the sense of self-as-object. This involves the inhibition of both the sense of self as a temporally extended object and higher–order, meta-cognitive forms of self-conceptualisation. Nevertheless, we stress that self-awareness is not entirely lost in flow. Rather, it is pre-reflective and bodily. Our approach to bodily-action-centred phenomenology can be applied to similar facets of seemingly agentive experience beyond canonical flow states, providing insights into the mechanisms of so-called selfless experiences, embodied expertise and wellbeing.

## Introduction

A flow state is an “almost automatic, effortless, yet highly focused state of consciousness” (Csikszentmihalyi, 1997, p. 110) that manifests in the experience of skilled experts during their completion of a given, complex task (Nakamura and Csikszentmihalyi, 2009, 2014). It is often said that flow engenders a loss of self-awareness (Abuhamdeh, 2020; Kotler et al., 2022), although different conceptualisations of this phenomenon abound in the flow literature (cf., Keenan et al., 2000, p. 338; Strawson, 2000). For example, Gold and Ciorciari (2020, p. 3) claim that, in flow, individuals lose “the awareness of themselves”; Nakamura and Csikszentmihalyi (2014, p. 20) state that flowing individuals experience “a loss of themselves as a social actor”; Shepherd (2022, p. 970) speaks of a “loss of reflective self-consciousness”; and Van der Linden et al. (2021, p. 1) refer to “low levels of self-referential thinking” in flow. Furthermore, notwithstanding these definitional issues, it is not immediately clear why flow should lead to any attenuation in the sense of self. Indeed, a flow state seems to be the optimal environment for an organism to exercise its skilful, *predicted* schema of action. Subsequently, it is at least plausible that psychological models which root the key features of a sense of self – namely, a first-person perspective, as well as a sense of possession and agency – in sensorimotor contingencies and their integration should predict that flow should *accentuate* the sense of self, not *attenuate* it, given that such integration is achieved in flow (Wolpert et al., 1995; Blakemore and Frith, 2003; Friston et al., 2010; Christoff et al., 2011; Friston, 2012a; Adams et al., 2013).

In this paper, we aim to resolve both issues: (i) *what* type of self-awareness disappears in flow and (ii) *why* flow causes such modulations to self-awareness. To do so, we must first investigate what self-awareness means. Following the self-model theory of subjectivity (SMT) (Metzinger, 2003, 2004, 2005a, 2008, 2009, 2013a,b, 2015, 2017, 2020, 2024), one can distinguish between several levels or layers of conscious self-experience, ranging from minimal phenomenal selfhood (MPS) and associated concepts like the pre-reflective *bodily subject* (Merleau-Ponty, 1962; Zahavi, 1999, 2005; Gallagher, 2003; Legrand, 2006, 2007a,b; Blanke and Metzinger, 2009; Limanowski and Blankenburg, 2013) to high-level, reflective self-representations implicit in a so-called *epistemic self model* (ESM) (Metzinger, 2015, 2017; Dołȩga, 2018).^1^

In this paper, we will define the ESM as a level in the phenomenal space of selfhood, which, when identified with, yields the sense of a ‘knowing self,’ i.e., a (pre-reflective) self that seems to stand in an epistemic relation with (knowing, thinking about, understanding) a world of stable epistemic value, which might include itself-as-object (Metzinger, 2015, 2017, 2024). Furthermore, epistemic selfhood is typically tied to agency, such that this epistemic directedness is frequently felt to be purposive, controlled and goal-driven, yielding not only an ESM but an epistemic agent model (EAM) (Metzinger, 2013a, b, 2015, 2017, 2024). Note that we take the EAM to be a subset of – and not equivalent to – the ESM.^2^

Crucially, a broad category of *phenomenal self-reflection* is constitutive — albeit not necessarily defining, see below — of the ESM. In other words, knowing selves can know themselves. For example, the act of (counterfactual) planning, a quintessential capacity of a seemingly knowing self, often engenders a sense of self as a temporal object which has been projected (by itself) into the future, as well as the past.^3^^,^^4^ For the sake of simplicity, we label this the *temporally-extended-self-as-object*. Crucially, we do not make the strict claim that *all* acts of mental time-travel – or “autonoetic” consciousness – *necessarily* yield the sense of self-as-object (Tulving, 1985; Wheeler et al., 1997; Klein, 2016). For example, it is at least plausible that in recalling what I ate for dinner last night, awareness of self is only given in a pre-reflective fashion.^5^ It is less clear that one can plan without reflectively experiencing oneself as a thing. More importantly, however, it *is* the case that certain instances of planning and recollection *do* involve the sense of self-as-object. For example, when I think of my holiday next year, I often think *of myself* on the beach; that is, myself is an intended object. Thus, if autonoetic consciousness is inhibited, then the reflective self-awareness that often accompanies it will be inhibited too.

Furthermore, organisms endowed with ESMs are generally capable of more overt self-reflections, including a form of abstract, propositional self-conceptualisation — i.e., “I am a thing” — whereby the self is experienced as a historicised object (Metzinger, 2015, 2017; cf. Limanowski and Friston, 2018). We label this the *conceptually-represented-self-as-object*. Further, according to the SMT, self-experience can vary both between organisms (e.g., some organisms only possess a pre-reflective, minimal sense of self, whereas others have reflective self-models) and, crucially, within an organism over time (i.e., one can experience different forms of phenomenal or non-phenomenal self-awareness depending on whether one is sleeping, in a coma, or awake).

As noted above, it is unclear, given former analyses of flow states, whether we should interpret the attested modulations in the phenomenal sense of self in flow states as indicating changes in the self-reflective aspects of the ESM (and, if so, which of its elements) or changes in a more basic aspect of phenomenal selfhood, which manifests as an intransitive, pre-reflective self-awareness (*ipseity*), whereby experiences (structurally) have a certain “for-me-ness”^6^ a first-personal givenness, a sense of a recipient of those experiences who is not herself an object of awareness (i.e., a dative, not an accusative, of experience; cf., Merleau-Ponty, 1962; Shoemaker, 1968; Hurley, 1998; Panksepp, 1998; Zahavi, 1999, 2003, 2005, 2017, 2020; Damasio, 1999; Gallagher and Marcel, 1999; Gallagher, 2003, 2023; Metzinger, 2003; Legrand, 2006, 2007a,b; Thompson, 2007, p. 251; Williford, 2016; Guillot, 2017). In other words, previous work has not specified whether flow alters the consciousness of a self as it is the object of an experience (sense of self-as-object) or the consciousness of a self as it is the subject of an experience (sense of self-as-subject, or a non-objectifying self-acquaintance) whereby that experience – which might have as its object the intended self-as-object – is given through a first-person perspective and is felt to be *for* a subject who, herself, is not intended. Note that we purposefully refer to the *sense* of the self-as-subject and the *sense* of the self-as-object – as well as phenomenal self-models – in this paper, to avoid making any ontological commitments as to whether there *is* a self in any substantial sense, and thus whether self-reflexivity should narrowly refer to consciousness’ (non-objectifying) (self)awareness of itself, or more broadly to a personal entity which has that experience (Gurwitsch, 1941; Henry, 1963, 1965; Zahavi, 2005, 2020; Frank, 2022).

We can begin by noting that flow clearly inhibits the act of thematically *conceptualising* oneself as a distinct object. That said, its phenomenological influence goes beyond this one modulation of the self-model. Indeed, if the phenomenological consequence of being in flow was simply the prevention of *self-conceptualisation*, flow would hardly constitute an interesting psychological phenomenon, given the absence of such meta-cognition from most of our experiential lives (cf., Poellner, 2003; Metzinger, 2005a, p. 22; Thompson, 2007, p. 308, 312). We shall therefore propose that the environmental constraints associated with flow – which we shall discuss using the formalisms of active inference (Friston, 2010; Ramstead et al., 2022) – inhibits both forms of phenomenologically reflective self-awareness mentioned above, which we argue are constitutive – but not defining – of an ESM: not only the aforementioned higher-order self-conceptualisation, but also the self-reflective sense of being in a temporal landscape which differs from the present, an experience that frequently accompanies deep counterfactual planning (as well as recollective memory; Gilboa, 2004; Macrae et al., 2004). This second type of epistemic selfhood involves a sense of both pre-reflective self-awareness — i.e., I am (pre-reflectively) guiding my internal attention in an autonoetic fashion — and reflective temporality — i.e., I am an object in a remembered past and/or an imagined future — where both phenomenal features are lost in flow.^7^ Again, the fact that not all forms of autonoetic consciousness necessarily involves a reflective sense of self-as-object is *not* a critical issue. This is because flow inhibits all forms of autonoetic consciousness, which *includes* those instances where a self-as-object is directly intended. Thus, the existence of autonoetic consciousness devoid of the sense of self-as-object does not threaten the validity of our central claim: namely, that as a result of the constraints the flow context places on the embodied cognitive system, mental time-travel, as well as self-conceptualisation, is inhibited and, consequently, the emergence of reflective self-awareness is too.

Nevertheless, self-awareness is not absent in flow; rather, it is pre-reflective (identification free) and bodily (Sartre, 1956; Wittgenstein, 1958; Merleau-Ponty, 1962; Husserl, 1989; Leder, 1990; Gallagher, 2003, 2005, 2023; Zahavi, 2005; Legrand, 2006, 2007a,b; Thompson, 2007; Solms and Panksepp, 2012). More specifically, pre-reflective bodily self-awareness in flow is both “performative” – i.e., the body is experienced as a subject-agent – and “transparent” – i.e., awareness of the world is given in a bodily mode (Gallagher, 2005, p. 74; Legrand, 2007b). Given this, it would be misleading to say flow eliminates all sense of a knowing self, given that pre-reflective bodily self-awareness in flow is marked by a (pre-reflective) sense of (embodied) familiarity, expertise and control (Metzinger, 2017; Lavoie et al., 2022, section 2.5). It is for this reason that we focus on the elimination of the two particular forms of epistemic selfhood that entail objectual self-reflection, rather than epistemic selfhood wholesale. As mentioned above, the self-awareness embedded in the flow experience is notably pre-reflective and can be aptly described as a maintained sense of a bodily self-as-subject that is experienced correlatively with (and not separately from) the world but is never transformed into an intentional object of awareness (i.e., a self-as-object) (Legrand, 2007a,b; Zahavi, 1999, 2005). However, this sense of self-as-subject also includes the (non-objectified) embodied sense of skill and know-how that, under Metzinger (2013b, p. 8; cf., Metzinger, 2013a, 2015, 2017) definition of the EAM^8^, might seem to belong to an epistemic agent who, nevertheless, does not intentionally take themselves to be so. Furthermore, there are other cases of epistemic behaviour which do not involve the sense of self-as-object (cf., see text footnote 7).

It is, thus, the dividing line of pre-reflective (versus reflective) self-awareness – or sense of (bodily) self-as-subject (versus sense of (bodily) self-as-object) – that is crucial to understanding flow and distinguishes it (and other experiences) from more quotidian life, not the distinction Metzinger draws between the ESM and the MPS, the latter of which, we take in this paper, for simplicity’s sake, to be isomorphic to pre-reflective self-awareness (albeit recognising that this act of identification masks important differences between how the two constructs have historically been presented, cf., see text footnote 21; Blanke and Metzinger, 2009; Limanowski and Blankenburg, 2013; Metzinger, 2013b; Zahavi, 2020; Kim and Effken, 2022; Gallagher, 2023). Indeed, given this presuppositional isomorphy, we can claim that some MPSs belong to ESMs, and even EAMs, as in flow. Nevertheless, Metzinger’s SMT (2003, 2004, 2008, 2009) and the spectrum of self-awareness it proffers is still a useful frame through which to discuss this difference, given the fact that self-reflective awareness is always an epistemic act (putatively associated with an ESM), even if not all epistemic acts entail self-reflective awareness (Metzinger, 2015).

In addition to providing this account of self-awareness within flow states, we will explain some of flow’s other defining characteristics using the formal computational approach of active inference (Ramstead et al., 2022). This discussion will analyse the sense of cognitive effortlessness felt by those in flow, its intrinsically rewarding (autotelic) nature, whether it induces learning and how it relates to boredom.

Finally, it is worth noting that flow states have not been studied in great depth within the active inference framework.^9^ Given that active inference is often proposed as a grand unifying theory of neurocognitive functions, applying its framework to an apparently universal cognitive state like flow is highly apt (Clark, 2013; Csikszentmihalyi and Asakawa, 2016). This computational approach is particularly needed in the domain of flow states, where the majority of research has been qualitative. Furthermore, understanding the mechanisms underlying flow and the concomitant modulation of certain aspects of self-awareness within it might afford us further insight into the functional nature of conditions involving more dysfunctional forms of selflessness, such as depersonalisation, of which different active inference models have been proposed (absent a theoretical synthesis) (Seth et al., 2011; Gerrans, 2019; Ciaunica et al., 2020, 2022; Deane et al., 2020). Finally, it has been shown that individuals who experience flow more frequently have greater self-esteem and a higher life satisfaction than those who do not (Tse et al., 2020, 2021). Thus, naturalising the mechanisms of flow in terms of active inference has implications for research exploring well-being which are beyond a mere contrast with cognitive disorders like depersonalisation.

### Flow states: an overview

The term *flow state* typically refers to the cognitive state of heightened focus on and absorption in a task over which one feels a certain effortless control. Furthermore, to enter flow, an organism – sometimes referred to as an “agent” in the literature (cf., Shepherd, 2022; Bartholomeyczik et al., 2023; Hackert et al., 2023) – must have a sufficiently developed skillset to match the demands of a task (the so-called *balance hypothesis*; Csikszentmihalyi, 2003; Keller et al., 2011; Fong et al., 2014; Kennedy et al., 2014; Harmat et al., 2015; Baumann et al., 2016; Tozman et al., 2017). It is worth mentioning here that any use of the word “agent” in this paper should *not* be taken to imply an ontologically primitive entity in possession of its own properties, states and processes. Rather, when we do use the term, it is to naturalise our narrative. It should thus be considered shorthand for an individual organism’s embodied cognitive system from which action appears to emanate and in which perception appears to occur, without making the axiomatic presupposition that numerically demarcated “agents” really exist in this world. In general, we have shied away from the use of the word “agent’ because of the ontological commitments it implies, opting instead for the more neutral terms of “organism,” “embodied cognitive system,” “predictive system” and “individual,” recognising, nevertheless, that the word is frequently used in active inference and flow states papers, notwithstanding its connotations (e.g., Corcoran et al., 2020; Fountas et al., 2020; Matsumura et al., 2023).^10^

Several other elements of flow are worth explaining in further detail. Firstly, it involves a distortion of temporal experience, such that individuals in flow report time passing quickly (Rutrecht et al., 2021). This phenomenal quality is not exclusive to flow, although flow might offer a paradigmatic case through which to analyse the speed of time’s subjective passage (cf., Parvizi-Wayne, 2024a). Flow activity is also said to be intrinsically rewarding, or autotelic, such that it is undertaken for its own sake (Csikszentmihalyi, 1990; Jackson, 1996). For example, in a qualitative investigation of flow states within a group of contemporary dancers, Łucznik et al. (2021) highlight the positive experience of their participants, as illustrated by the following quotation:

*Dancer C:*

*“It is a good feeling; I really enjoy it. It creates in a way more space for me; I feel free.”*

This study also provides evidence for the claim that flow states yield a sense of effortless control, which, notably, seems associated with a lack of deep, propositional planning:

*Dancer A:*

*‘Flow is when I dance and everything that happens in movement happens naturally. That I do not need to think a few steps ahead: ‘Now I do this or that.”*

*Dancer B:*

*‘I can surprise myself, I can find myself in the places like, I do not know how I get here and I do not necessarily know how to get out of there.*

These quotes, as well as those below from Csikszentmihalyi (2014, p. 139) and Csikszentmihalyi (1975, p. 43), also point to the loss of the sense of self-as-object in flow, for which this paper will provide a computational account.

*An expert rock climber:*

*You are so involved in what you are doing, you aren’t thinking of yourself as separate from the immediate activity… you do not see yourself as separate from what you are doing…*

*Another climber:*

*It’s like when I was talking about things becoming ‘automatic’… almost like an egoless thing in a way– somehow the right thing is done without… thinking about it or doing anything at all… It just happens… and yet you are more concentrated.*

According to Csikszentmihalyi (2014, p, 138), this experience constitutes an action-awareness merger, whereby one who is in flow “is very aware of one’s actions, but not of the awareness itself,” a feature of flow which he holds to be distinct from the modulations to self-awareness (cf., Nakamura and Csikszentmihalyi, 2014). However, as Shepherd (2022, p. 13) points out, it is “unclear to what degree these can be kept separate”; indeed, the very example Csikszentmihalyi (2014, p, 138) gives of the action-awareness merger in rock-climbing seems to involve a loss of reflective self-consciousness rather than a loss of reflective-consciousness *per se*.

In fact, the very act of reflecting on consciousness generally – although it is unclear what Csikszentmihalyi (2014) means by this – seems to involve a reflective *self*-consciousness, such that I recognise that *I* am having this experience. Note that, incidentally, Csikszentmihalyi et al. (2005) conflate the action-awareness merger and the modulation to self-awareness. We, thus, propose that what has been termed the action-awareness merger just *is* the experience of pre-reflective bodily self-awareness – both its “transparent” and “performative” aspects – in flow. Equally, one might wish to say that the action-awareness merger just *is* the loss of reflective self-awareness. In any case, we hold that the loss of reflective self-awareness in flow is the result of the specific precision weighting mechanisms and the curtailed planning horizon that flow engenders. These constraints lead the organism to maximise the pragmatic value at hand through optimal (bodily) performance, yielding what Łucznik et al. (2021, pp. 22–23) term “body-thinking; solving problems in a non-propositional way in which the reflective processes and explicit knowledge were limited through full attention on the dance and body.” This last point – that flow states are entirely absorbing – is evidenced by the following quotation, from Swann et al. (2019). Note here the allusions to the lack of autonoetic consciousness and the positive valence associated with flow:

*Yoga 1:*

*“You’re completely absorbed in the moment. So you are not in the past, you are not in the future, so I think that gives you peace of mind.”*

### An active inference account of flow states

Our aim in this paper is to give a thorough, formal account of these phenomenological markers of the flow state from an active inference perspective. Active inference is a process theory which seeks to elucidate how complex entities such as humans persist in ever-changing environments (Limanowski and Blankenburg, 2013; Seth, 2013; Hohwy and Michael, 2017; Friston, 2018; Limanowski and Friston, 2018, 2020; Deane, 2020; Deane et al., 2020; Ciaunica et al., 2022). It is therefore a corollary of the free-energy principle, which states that if something persists through time, it can always be described as instantiating a statistical (generative) model of its environment, whereby the internal states of that model appear to be parametrising Bayesian beliefs about the external states. This can be further cast as the minimisation of variational *free energy* (VFE), an information-theoretic term which acts as an upper bound on surprisal or Shannon self-information: the negative log probability of some system’s states, given that system’s constitution (Friston, 2009, 2010, 2019; Kirchhoff et al., 2018; Ramstead et al., 2018, 2023). Note that, along these lines, self-organising systems will look as if they are actively trying to seek out evidence for the model that their existence implies and are therefore often said to be *self-evidencing* (Hohwy, 2016). In systems possessing a self-model (Metzinger, 2004), this can be taken as actively confirming that “I exist” (Limanowski and Blankenburg, 2013; Limanowski and Friston, 2018).

In cognitive creatures like ourselves, it has been proposed that free energy minimisation is achieved through a hierarchical predictive coding scheme^11^, in the brain and body, whereby such systems hold expectations of the states they find themselves in (their Bayesian priors) at different levels, such that “higher” levels — which model slower, more generalised flows — constrain and contextualise faster, lower level dynamics. These expectations are then used to generate predictions that are either corroborated or violated by incoming sensory data (Friston et al., 2010, 2017a; Clark, 2013, 2015; Pezzulo et al., 2018). Faced with a discrepancy between predictions and sensory observations (i.e., prediction errors), organisms like us are not at the behest of the environment, faced only with the option of updating our model to achieve a better fit (perceptual inference). Rather, we can *act* upon the world to change it so that the sensory samples, engendered by our behaviour, better accord with our prior expectations (active inference). Indeed, this active strategy is the only viable option in certain contexts: given interoceptive data which signal hunger and diverge from a prior expectation to be satiated, self-organisation can only be maintained by acting upon the world — e.g., by releasing insulin (an autonomic action) or eating (a motor action) — to return the organism back to its homeostatic set-point (i.e., characteristic or preferred state). Active inference can also take the shape of prospective action, whereby an organism, capable of deep temporal planning as inference, engages in future (expected) free-energy minimising (EFE) behaviour in anticipation of upcoming demands which would lead to dyshomeostatic outcomes (Sterling, 2012; Pezzulo et al., 2015, 2018; Barrett, 2017; Corcoran and Hohwy, 2018). This has been described in terms of allostasis. To provide a simple example, if I look outside and see that it is raining, I can plan ahead and ensure that I take an umbrella with me when I venture to the shops, which, in turn, reduces the VFE that I would have encountered if I had not planned ahead.

A final addendum to this background description ought to be made. Thus far, we have only touched upon the first-order action-perception cycle through which humans and other complex organisms self-evidence. Now, we add a second level to these predictive dynamics, namely that of precision (Friston, 2012b; Clark, 2013; Parr et al., 2018). Precision is technically the inverse dispersion (e.g., variance) of a probability distribution and can be understood in a metacognitive sense as a belief *about* beliefs. However, it is worth clarifying that here we are not talking about the folk-psychology notion of explicit, propositional beliefs about worldly states. Rather, in the context of active inference, we are talking about subpersonal *Bayesian* beliefs. Thus, the term “beliefs” should be taken solely to mean sets of (Gaussian) probability distributions, unless indicated otherwise. Indeed, there are times when an organism’s propositional beliefs do at least correspond with the Bayesian beliefs that define the type of thing that organism is (Smith et al., 2022). This is the case, for example, in instances of propositional self-conceptualisation: for me to explicitly believe that I am the type of thing that I am, there must be a Bayesian belief encoding this proposition as a type of preferred sensory outcome of the mental action of me thinking it. Furthermore, in possessing such a belief, there will always be a Bayesian belief that could describe me as the type of thing that has that belief. However, it may not be the case that I actually *am* the thing that I explicitly *believe* that I am, indicating a discord between the Bayesian beliefs that describe what I am and those that describe the higher-order propositional beliefs I possess (cf., self-deception; Pliushch, 2017; Marchi and Newen, 2022). In any case, it would be a mistake to conflate the mathematical level of description with the psycho-philosophical one.

Attention has been associated with the optimisation of precision weighting of the likelihood mapping within the active inference framework, whereby precision must be both estimated and deployed (Feldman and Friston, 2010; Mirza et al., 2019; Parr and Friston, 2019; Parvizi-Wayne, 2024b). However, it is important to note that one can ascribe precisions to other beliefs encoded by the generative model, including the precision of beliefs about how the world evolves, the precision of the prior expectations over sensory outcomes and the precision associated with the beliefs about policy selection. Inferring and performing actions over these second order beliefs has been offered as a computational account of mental action (Limanowski and Friston, 2018; Sandved-Smith et al., 2021). This broader account of variety second-order beliefs is required for the account that follows.

With this framework in place, we will now outline a computational model based on active inference that we believe explains the constraints flow states put on the free energy minimising generative model such that it evinces the phenomenality described above. The relevant and core characteristics of a flow context which entail these changes to the embodied cognitive system are (i) the learned expectations the person has about how the situation will unfold that result from extensive training and (ii) the challenging nature of the activity.

**Figure 1:**
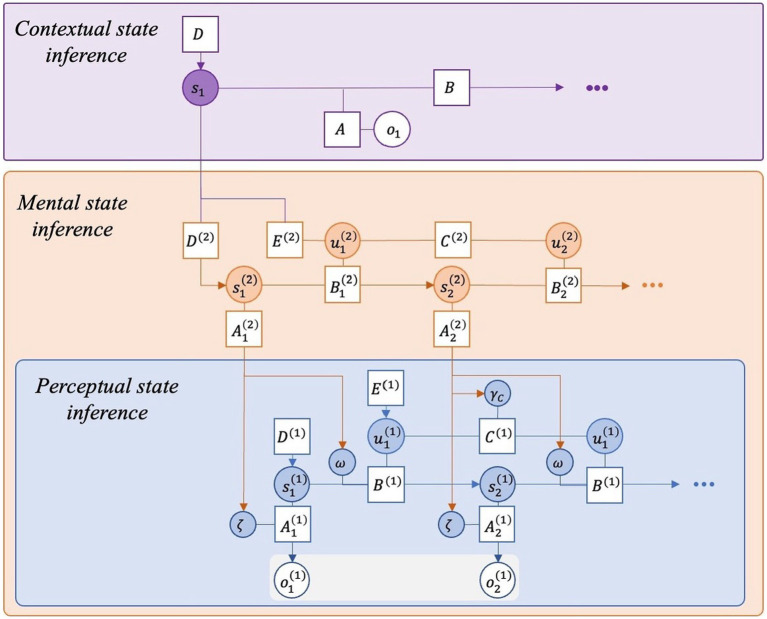
A hierarchical generative model of contextually cued behaviour (such as flow). This diagram depicts a hierarchical Bayes graph representing the inferential architecture we propose is responsible for the experience of flow. Some dependencies (i.e., edges) have been omitted for clarity (e.g., the dependency of initial perceptual states on mental states). Shaded circles represent inferred beliefs about states *s* and actions *u* given observations *o* and the parameters of the generative model in square boxes (A–E). The parameter A refers to the likelihood mapping – that is, the probability of making an observation *o* given a state *s*. B refers to beliefs about how states transition into others. C refers to prior beliefs (preferences) about sensory outcomes. D refers to beliefs about the initial state prior to any observations. E refers to priors over policies – what the organism would normally do, independent of the EFE in the current situation. This generative model exhibits two forms of hierarchical depth. First is *a conceptual depth* between the purple and orange levels in which the higher-level states initialise the state and policy at the level below (i.e., contextualise courses of action at the lower level). Second is a *parametric depth* between the orange and blue levels in which the higher-level states parameterise the precision weighting of beliefs encoded by the parameters of the level below. First the individual infers themself to be in the flow related context (contextual state inference at the purple level). This serves as a contextual cue for the deployment of learned prior beliefs about mental states and policies (i.e., ‘habitual’ mental actions). The policy selection that subsequently unfolds on the orange level results in multiple precision deployments on the lower (perceptual) level, increasing the precision of the likelihood mapping (ζ), the precision of the transition mapping (ω) and the precision of preferences (γ*_c_*). Note that this graph is limited in its capacity to show the shallowness of the planning involved in the deployment of physical actions in flow states. It, thus, does not sufficiently capture all the belief dynamics involved in flow; rather, it is a model of the inferential architecture underpinning contextually cued behaviour, notwithstanding the fact that not all contextually cued behaviours yield extremely high precision weight over ζ, ω and γ_*c*,_ as in flow.

When the person returns to the flow-inducing activity they have been practising they will infer that they are back in that familiar context. This contextual inference then provides a cue for a number of associated beliefs. Firstly, the repeated training means that the context triggers a high precision weighting over the beliefs about the impact of actions in terms of how latent states will transition: i.e., “I am confident about what should happen if I perform this action in this context.” In the partially observable Markov decision process (POMDP) schema utilised in discrete state space active inference models, these beliefs are encoded within the so-called B tensor (Friston et al., 2017b; Da Costa et al., 2023). Secondly, having experienced the situation many times, the context will also trigger precise expectations (C tensor) about sensory outcomes: i.e., “I am confident about what I should observe in this context.” The modulation of these precision weights is achieved via mental action selection that is cued by the familiar context (see Figure 1). The contextual inference furnishes priors over mental states and policies (higher order D and E tensors respectively). Priors over policies (E) can be thought of as ‘habitual policies’ given a particular context, hence the precision weighting deployments are triggered by the engagement of *mental* policies that are habitualised with training. Note that this does not imply that habituation is involved in the execution of the overt motor actions performed during flow.

Together these two second-order precision beliefs will result in action selection dominated by pragmatic value. The individual has high prior expectations (i.e., preferences) about the states it expects to occupy and low uncertainty about how to fulfil those expectations. The imperative to minimise EFE therefore drives the person to capitalise on this predictably rewarding situation, by performing the actions demanded by the task, rather than engaging in epistemic behaviour, such as the resolution of present uncertainty (information gain) or novelty-seeking, which can be directed either at the updating of model parameters or broader structure learning (Botvinick and Toussaint, 2012; Friston et al., 2015, 2016; Mirza et al., 2016; Kaplan and Friston, 2018; Smith et al., 2020). Indeed, the minimisation of EFE in active inference can be described in terms of both epistemic and pragmatic affordances (cf., Equation 1).

Equation 1: EFE Equation.

Gu=EQulnQsτ|u−lnQsτ|oτ,u−lnpoτ|c=−EQulnQsτ|oτ,u−lnQsτ|u︸expectedinformationgain−EQu[lnpoτ|c︸expectedvalue]=DKLQoτ|u||Poτc︸risk−EQulnQoτ|sτ,u︸ambiguity

Here, G stands for the EFE for a given path or policy, *u*, where the goodness of a policy is scored by the negative EFE associated with it. What this equation shows is that EFE can be decomposed into the maximisation of Bayesian surprise, cast as the KL divergence between posterior and prior beliefs about future states conditioned on an action policy and pragmatic value, conditioned on preferred observations (Itti and Baldi, 2009; Friston et al., 2015, 2016; Kaplan and Friston, 2018). In this equation, prior preferences *p*(*o_t_* | *c*) are conditioned upon model parameters (*c*) that encode the outcomes that characterise the kind of organism in question. In effect, these priors underwrite the preferred outcomes the organism will plan towards. Crucially, high precision weight over this model parameter, as well as the state transitions that will unfold as a result of my action (B), drives the embodied cognitive system towards the exploitation of the pragmatic affordances at hand. High precision weight over the beliefs encoded within these parameters is only possible because the cognitive system has encoded a belief that there is no posterior uncertainty associated with the action policy which conditions these beliefs. In other words, the individual subpersonally recognises that carrying out this specific action will not yield information gain through new observations. This thereby reduces the imperative underlying action to the maximisation of pragmatic value. Indeed, if there was ambiguity about the outcome of action, that uncertainty would need to be resolved – in a manner which would involve a degree of planning - before pragmatic action could occur. As mentioned above, repeated training has served to eliminate any such doubts. Note that the organism does not ‘choose’ pragmatic or epistemic action *per se*; rather, they always select the action that minimises EFE, which can be expressed in terms of pragmatic and epistemic value. In short, minimising EFE subsumes the dual aspects of Bayes optimality; namely, maximising expected information gain in accord with the principles of optimal experimental design (Lindley, 1956) and maximising expected value, in accord with Bayesian decision theory (Berger, 1985). Interestingly, this means that pragmatic value and attending affordances acquire the same currency as epistemic value; namely, natural units (cf., bits of information with binary logarithms).

So far, this describes a situation that is often encountered, whereby the reward is high and the path is clear: e.g., I’m hungry and dinner is served. Using the formalism of a hierarchical Bayes graph (Figure 1), we can model the belief architecture of an individual’s generative model in such a situation, i.e., one in which a contextual cue prompts a mental action policy that results in high precision over lower-order beliefs about state transitions contingent on action policies (B tensor) as well as the sensory outcomes of those policies (C tensor).^12^ As mentioned above, this is quintessential of the flow state.

However, the challenging, complex nature of the flow-inducing activity creates two further constraints on active inference, which drive the phenomenology that defines the flow state. Both constraints arise from the fact that minor fluctuations in the situation must be met by immediate and appropriate motor actions (Klasen et al., 2012; Huskey et al., 2018a). In the case of the violinist, a slight deviation from the correct note must be corrected instantaneously; in the case of the surfer, a subtle change in the pitch of the wave face demands a quick shift of balance.

The first consequence of this is that the person’s attention must be highly focused on the incoming sensory data, in order to quickly detect these important changes. If the challenge is appropriately scaled to the individual’s ability, this will require most, if not all, of their attentional resources, since the task at hand is inferred to be sufficiently [although manageably (cf. Hohwy, 2022)] volatile such that any distraction will prevent the (minimal) planning and actualisation of the next action policy needed to continue the flow state (cf., Csikszentmihalyi, 2002, p. 54; Dietrich, 2003; Dietrich and Sparling, 2004). Computationally, this amounts to a high precision weighting on the likelihood mapping between sensory evidence and inferred perceptual states, encoded within the A tensor in POMDP schema. The experience is therefore dominated by presently incoming, action-relevant sensory data, which inhibits all forms of mental time-travel, including planning and retrospection (Klasen et al., 2012; Yoshida et al., 2014; Katahira et al., 2018; Eschmann et al., 2022).

Crucially, this constraint distinguishes flow states from more quotidian examples of pragmatic behaviour which, in and of their own execution, do not yield reflective self-awareness and therefore might seem similar to flow states. For example, when I grip my coffee cup to drink from it, I know the exact consequences of my action and, thus, need not resolve any uncertainty nor plan ahead extensively. Thus, with respect to my act of gripping *alone*, the mechanisms underlying my action are analogous to those when I am playing a violin concerto in flow. As expected, a similar phenomenology emerges, whereby I do not depict myself as an entity akin to the cup in our shared objectivity, but the gripping is consciously expressed in a pre-reflective bodily mode (cf., Merleau-Ponty, 1962; Dreyfus, 1990, 2002, 2014; Thompson, 2007; Heidegger, 2010; Tufft, 2022).^13^ In other words, both in violin-playing and cup-gripping, the motor system employs procedural knowledge, or an implicit knowledge of *how* to do something (know-how), to act on affordances, possibilities for action offered to the organism from its environment (Dewey, 1922; Ryle, 1949; Gibson, 1979; Friston et al., 2012; Bruineberg and Rietveld, 2014; Kiverstein and Rietveld, 2015; Bruineberg et al., 2018; Scholz et al., 2022; Pitliya and Murphy, 2023). However, canonical flow states, as in the violin concerto, *are* different from instances of what Dreyfus (1990, p. 104) refers to as *background coping*^14^ or what we might describe as *everyday flow states* (e.g., less complex actions such as cup-gripping or walking through doors) (cf., Collins and Evans, 2007; Bergamin, 2017). In part, this is because canonical flow states often last longer (Rutrecht et al., 2021). More importantly, everyday flow does not require the wholesale deployment of attention, because the action in question (e.g., cup-gripping), does not warrant a high likelihood precision weight encoded in the A tensor. Consequently, the experience of everyday flow can be *supplemented* by epistemic cognition: I can plan what I will eat for lunch whilst reaching out for my coffee cup (Bergamin, 2017). This is different from flow states, where the cognitive system entirely distributes attention *outside* itself, across the body’s actions and their sensory consequences, yielding the unique action-centric phenomenology comprising flow and inhibiting the emergence of the sense of self-as-object (Dietrich, 2004).

Note too that the wholesale attentional absorption that flow engenders makes it antithetical to the phenomenon of “choking under pressure,” defined as “an acute and considerable decrease in skill execution and performance when self-expected standards are normally achievable, which is the result of increased anxiety under perceived pressure” (Mesagno and Hill, 2013, p. 273; cf., Masters, 1992; Oudejans et al., 2011; Gröpel and Mesagno, 2019). We propose that choking is likely the result of an accumulation of free energy with respect to precise preferences located at “higher” levels of the generative hierarchy than those in which the (mere) preferred sensory outcomes of action are encoded. For example, if I am so concerned about my legacy and recognise that it is contingent on me scoring the upcoming penalty in football, I will be unable to enter into a flow state because my attention will be enslaved by that higher-level, narrative preference; i.e., the manner in which it is under threat, rather than the actual task dynamics (Parvizi-Wayne, 2024b). Given the complexity of flow contexts and the need for absolute attention in order to rollout regimes of adaptive expert action, any such distraction will engender decreased performance, which, in turn, will provoke more free energy with respect to the higher-order preference, which will lead to even worse performance and so on, spiralling into the feedback loop that typifies choking.

The second consequence is that whilst the person has high confidence in their beliefs about the effects of their actions (i.e., they are well trained), the ever-changing nature of the task means that these beliefs must remain temporally *shallow* (Pike, 1974; Berliner, 1994).^15^ In other words, there is a recognised volatility of the environment that precludes temporally deep action planning (Dietrich, 2004). Interestingly, this does not provoke an epistemic policy in order to resolve the uncertainty about the future, because the person has learned that they will know what to do, when the next moment arises – i.e., there is a higher order belief that, although significant environmental volatility is at hand, such volatility is manageable (Hohwy, 2022). More precisely, each new moment provides the contextual cue for the deployment of highly precisely weighted beliefs about the consequences of action, as described above, giving rise to unambiguous action selection and perpetuating the flow state moment by moment. As a result, the temporal depth of the action model contracts, so that it approaches the present moment. We propose that these two specific features of flow states – namely, the need for wholesale attention on the unfolding task and the inhibition of deep temporal planning – work *symbiotically* to yield the unique phenomenology of flow. As will be elucidated more thoroughly below, we hold that this diminishes the subjective experience of being an epistemic agent in multiple ways.

Interestingly, our proposal — that flow involves the contraction of the planning horizon — aligns with other contexts in which EFE, as a future-pointing construct, converges towards an equivalence with VFE, which the organism must minimise *now* to maintain its existence. For example, Safron (2020, p. 37) notes that psychedelic and meditative experiences engender the downregulation of the default mode network (DMN), which has been implicated as “the basis for imagination of counterfactual possibilities [and] mental time travel” among other capacities, and is constituted primarily by the ventral medial prefrontal cortex, the dorsal medial prefrontal cortex, the posterior cingulate cortex and precuneus (cf., Dietrich, 2003; Brewer et al., 2011, 2013; Carhart-Harris et al., 2012; Hassabis et al., 2014; Raichle, 2015; Davey and Harrison, 2018; Graziano, 2019; Smigielski et al., 2019; Li et al., 2020). This makes the down-activation of the DMN a plausible correlate of the diminished propensity to plan in flow states and deep meditative and psychedelic experiences (Spreng et al., 2010; Gerlach et al., 2011, 2014; Palhano-Fontes et al., 2015; Lutz et al., 2016; Hasenkamp, 2018; Millière et al., 2018; Deane, 2020; although see Safron, 2021b, p. 11). Indeed, brain imaging studies have shown that the activity of the DMN is also lowered during flow states, further reinforcing our proposal that there is an alignment between flow states and other altered states (Ulrich et al., 2014, 2016; Van der Linden et al., 2021). Furthermore, all three experiences are associated with the modulation of certain aspects of self-awareness, which, Deane et al. (2020) argues — in a manner similar to our own, see below — is rooted in a collapse of the temporal thickness of the generative model. Indeed, the DMN has been shown to be involved in self-referential processing (Northoff et al., 2006),^16^ overt self-reflection (Jenkins and Mitchell, 2011; D’Argembeau, 2018) and autobiographical memory retrieval (Gilboa, 2004; Cabeza and St Jacques, 2007). This leads us to tentatively suggest that there might be multiple varieties of flow state experiences, beyond canonical examples discussed in the flow literature and including those psychedelic and meditative (among others), unified in their constriction of the flowing individual’s temporal horizon and the subsequent phenomenological modulations to their sense of self (Dietrich, 2003).

In summary, we propose that the combination of training and the challenging nature of the task result in specific precision weighting allocations, as well as a restricted temporal horizon of action planning, which, as will now be shown, inhibit the emergence of the reflective self-as-object.^17^

### Self-awareness in flow

Thus far, we have offered a broad-brush account of flow states utilising the formal mechanisms of active inference. In order to explain how flow leads to the elimination of the reflective features of the ESM in greater depth, we take inspiration and insight from the substantial work that has focused on the emergence and maintenance of a sense of self within an active inference framework over the last decade or so. Indeed, this work has been centred around the construction of a phenomenal self, which can be defined as “the way you appear to yourself, subjectively and at the level of conscious experience” (Metzinger, 2004, p. 26), and can thus be distinguished from the notion of a *substantial* self, a putatively unchanging, ontologically independent entity from the brain/body. According to Metzinger (2004, 2005a, 2008), from which many active inference accounts of selfhood take inspiration (e.g., Limanowski and Friston, 2018; Deane, 2020, 2021; Ciaunica et al., 2022), the phenomenal self is generated by the neurocomputational mechanisms comprising a phenomenal self-model (PSM), a theoretical, representational entity which simulates and emulates^18^ the properties, states and outputs of its own system for itself, and whose contents just are the content of the conscious self. Crucially, the PSM putatively makes the representational outputs of its simulation/emulation globally available to its system (us), whilst, in almost all cases, hiding the underlying neurocomputational processes (i.e., its representational carrier). In other words, the vast majority of conscious representations are “transparent” to the organism, such that all that is experientially given is the representational content and not the vehicle. Thus, the cognitive system identifies itself with the content of its PSM, blind to the fact that the content “is an abstract property of the concrete representational state in your brain” (Metzinger, 2005a, p. 13; cf., Metzinger, 2003; Himma, 2005; Limanowski and Friston, 2018). Note that the use of the term “transparent” in Metzinger’s SMT – as a graded property of phenomenal representations – is different from its use in Legrand’s account of the “transparent body” – the bodily mode through which the world is experienced. In sum, according to Metzinger’s SMT, self-modelling is a complex, dynamical process, grounded in physiological mechanisms, which, nevertheless, afford a subjective, first-person perspective directed at the world(−model) via a modelled intentionality relation (cf., phenomenal model of the intentionality-relation; Metzinger, 2000, 2003, 2004, 2005a, 2008, 2009). The critical, additional claim that the active inference framework makes is that the mechanisms underwriting both the PSM and its representational targets are inferential and can be described as if they are fundamentally directed towards the goal of sustained self-organisation (Deane, 2021).

Under the active inference framework, the self has been cast as the globally available partition of a system’s “best guess” at the underlying cause of multi-modal sensory information and is encoded at “higher” levels of the hierarchical, predictive system that the organism embodies (Friston, 2012a, 2018; Limanowski and Blankenburg, 2013; Apps and Tsakiris, 2014; Hohwy and Michael, 2017; Deane, 2020, 2021; Deane et al., 2020; Limanowski and Friston, 2020). *Prima facie*, this might seem puzzling, since we might be inclined to associate our observations with the apparently *external*, latent causes that engender them. However, it is important to recognise that a complex predictive system is capable of mapping causal chains and is thus able to infer that “I” caused the observed state in the first place. It is worth recognising that this inference is, indeed, an inference, which need not map onto any ontological primacy of the self as a causal agent in the world. This is nevertheless a likely inference for such an organism to make, since, in active inference, actions are the method by which the animate entity can bring about states which bring forth expected sensory outcomes (Friston et al., 2010). Thus, as Deane et al. (2020, p. 7) argues, “in order to act, then, the system implicitly infers its own ability to bring about the intended sensory consequences.” In other words, an arguably necessary concomitant of self-evidencing behaviour (e.g., homeostatic regulation) is a sense of an entity *for whom* that action is produced, a self that can actively find ever-new evidence for *its* own existence by changing the world (Friston, 2012a, 2018). As such, the self is unique (to itself) among all other objects in its inherent reflexivity, as it must, according to active inference, “maximise evidence for the hypothesis it entertains about itself” (Limanowski and Friston, 2020, p. 5).

Further, the sense of agentive control that accompanies selfhood can be explained via the fact that the sensory outcomes of self-generated actions are usually highly predictable, i.e., *I* know what it will feel and look like if *I* move *my* arm there (Fletcher and Frith, 2009; Synofzik et al., 2010; Voss et al., 2010; Seth et al., 2011; Clark, 2020). This distinguishes self-generated actions from worldly events, which can be highly unpredictable. What’s more, the observation of such events is often not spatiotemporally contiguous with any underlying prediction, unlike actions of the self, which, according to active inference, are causally preceded by predictions (Friston et al., 2010; Friston, 2012a; Adams et al., 2013). Thus, the organism is able to distinguish (re)afferent sensory signals which are self-generated (because they are matched with an internal prediction) with (ex)afferent signals that arise from the (non-self) environment, since, frequently, these cannot be matched with a prediction that temporally preceded them (Sommer and Wurtz, 2008). This functional distinction has implications for various facets of self-experience, ranging from psychophysical sensory attenuation to perceptual illusions; and failures in the underlying computations can lead to disruptions in those processes as in schizophrenia or hyperreflective ‘freezing,’ for example (Adams et al., 2013; Brown et al., 2013; Limanowski, 2017; Deane, 2020, 2021).

This active inference model of active self-evidencing and the implied sensory attenuation of self-generated sensations corresponds — somewhat^19^ — with earlier accounts which stress the importance of *self-specifying* processes of sensorimotor integration in the generation of self-awareness (cf., Von Holst, 1954; Wolpert et al., 1995; Blakemore et al., 1999; Blakemore and Frith, 2003; Legrand, 2006, 2007a,b; Legrand and Ruby, 2009; Christoff et al., 2011). Finally, earlier models have also recognised that interoceptive, homeostatic regulation, which underwrites a maintained bodily integrity, also engenders a functional self/non-self distinction. This is because reafferent-efferent loops are also embedded within the interoceptive system and therefore self-specify the body as an “agent” self-individuating against the backdrop of the non-self environment (Christoff et al., 2011, p. 3; Damasio, 1999; Parvizi and Damasio, 2001; Thompson, 2007; Craig, 2009; Seth et al., 2011; Seth, 2013). This is in line with our claims made above as well as the broader theoretical focus of active inference (Deane, 2021). That said, although we suggest that such properties of self-evidencing entities might plausibly underwrite their sense of self and agency, we also recognise that grounding the phenomenology of selfhood in active inference is an ongoing project which cannot be wholly achieved in this paper.

Furthermore, although the concepts are somewhat elided in the above paragraph, it is worth keeping in mind the distinction between the sense of the self-as-subject (i.e., pre-reflective self-awareness) and the sense of the self-as-object (i.e., reflective self-awareness), recognising, firstly, that different cognitive processes likely underwrite these distinct phenomena and, secondly, that phenomenologists frequently argue that the existence of pre-reflective self-awareness *presupposes the possibility for* higher-order, self-conceptualisation (Sartre, 1956; Merleau-Ponty, 1962; Flanagan, 1992; Bermudez, 1998; Edwards, 1998; Legrand, 2006; Legrand and Ruby, 2009, p. 20; although see Metzinger, 2024 for a potential third category: non-egoic awareness). To recall, awareness of self-as-subject is a pre-reflective self-awareness: the non-objectual sense of a subject for whom and to whom experience is given (Zahavi, 2005, 2020; Legrand, 2006, 2007a,b; Guillot, 2017). Conversely, awareness of the self-as-object is awareness of the self as an object in consciousness, thereby differing from the subjective experience in which it is intended (Legrand, 2007a, p. 586; Sartre, 1948). This includes the awareness of both the temporally-extended-self-as-object that generally emerges in planning and the conceptually-represented-self-as-object that is intended in moments of meta-conceptualisation.

Within the active inference framework, these different dimensions of self-awareness are often cashed out in terms of the temporal depth of the generative hierarchy from which a sense of self emerges (Deane, 2020, 2021; Friston, 2018). This, in turn, permits an association of computational and phenomenal self-models, according to which temporal depth maps onto phenomenological ‘thickness’ (cf. Metzinger, 2004; Ramstead et al., 2022). At a level of great phenomenological “thinness”^20^ emerges pre-reflective self-awareness or what might otherwise be described as the MPS (Blanke and Metzinger, 2009; Limanowski and Blankenburg, 2013; Metzinger, 2013b). This, according to Blanke and Metzinger (2009, p. 8), has three features: (i) identification with the body (ii) spatiotemporal self-location and (iii) a weak first-person perspective (a point of perspectival projection from within the body), yielding a mapping between the experiential centredness of our reality and the centredness, or origin, of our behavioural space (Metzinger, 2005a, p. 17). In line with Blanke and Metzinger’s (2009) emphasis on the connection between the body and the MPS, recent theorists, often working within the active inference framework, have ground (at least aspects of) the MPS in interoceptive inference (Friston, 2011; Seth et al., 2011; Critchley and Seth, 2012; Gu et al., 2013; Limanowski and Blankenburg, 2013; Seth, 2013; Suzuki et al., 2013; Barrett and Simmons, 2015; Seth and Friston, 2016).

This, in turn, corresponds to phenomenological claims suggesting that the MPS (i.e., pre-reflective self-awareness) is a fundamentally *bodily* phenomenon, whereby the bodily self is “lived through to the world” rather than transitively objectified (Merleau-Ponty, 1962; Husserl, 1973; Damasio, 1999; Depraz, 2001; Legrand, 2006, 2007a,b, 2010; Christoff et al., 2011, p. 139; Sartre, 1956, p. 328; Thompson, 2007).^21^ Here, bodily pre-reflective self-awareness emerges not as consciousness of a body which happens to be my own, but rather the non-intentional “consciousness of one’s body as oneself” (Legrand, 2006, p. 90), yielding the sense of a bodily self-as-subject for whom and to whom the experiential world, within which it is enmeshed, is given, or with which the world-as-object, which may include the body-as-object, is correlatively experienced (Legrand, 2007a,b, 2010). Following Legrand (2007b), we will call this more minimal sense of pre-reflective bodily self-awareness the experience of the “transparent” body. However, pre-reflective bodily self-awareness can also be “performative,” as Legrand (2007b) puts it. By this, she means that the body “is experienced pre-reflectively as a subject-*agent*” (Legrand, 2007b, p. 506, emphasis added). As will become clear, flow states involve a *forefronted* experience of the “performative body”, as well as the maintained sense of the “transparent body”.

It is worth mentioning that pre-reflective self-awareness can be described as a phenomenon even broader than pre-reflective *bodily* self-awareness or the sense of the *bodily* self-as-subject (cf., Metzinger, 2013b). Many philosophers in fact argue that it is an intrinsic aspect of consciousness; in other words, without pre-reflective self-awareness, there would be no phenomenal experience (Frankfurt, 1988, p. 162; Goldman, 1970, p. 96; Sartre, 1956; Zahavi, 1999, 2005; Legrand, 2007a; Kriegel, 2009; Kiverstein, 2020; although see Letheby, 2020, Millière, 2020, Winter et al., 2020, Deane, 2021, Laukkonen and Slagter, 2021, Metzinger, 2024). At this broadest level, pre-reflective self-awareness is not so much an awareness of a self, but what Gallagher and Zahavi (2023) call “the first-personal givenness of experience”, where the self is a mere “dative of manifestation”, to quote Zahavi (2005, p. 71; cf., Williford, 2016), and is inherent in the flow of experience (i.e., without a separate self-quale).

Our decision to focus on the sense of the bodily self-as-subject rests on the fact that this is how subjectivity is articulated most pronouncedly within flow states; nevertheless, this could be described as just a manifestation of the more fundamental subjective dimension of consciousness that is intrinsic to experience itself and can be present absent any bodily experience (cf., Metzinger, 2003, 2005b, 2013b; Blanke et al., 2008; Blanke and Metzinger, 2009; Windt, 2010). On the other hand, certain phenomenologists (cf., Merleau-Ponty, 1962; Husserl, 1973; Legrand, 2007b, 2010; Gallagher and Zahavi, 2008, 2023; Gallagher, 2023, pp. 172–173) seem to claim that pre-reflective self-awareness is always bodily (in the “transparent” sense), implying that our specific focus is not as exclusionary as it might first seem. Unfortunately, a more thorough examination of exactly what pre-reflective self-awareness involves - e.g., affect (Damasio, 1999; Colombetti and Ratcliffe, 2012), temporality (Husserl, 1973; Zahavi, 1999, 2003), or intersubjectivity (Ratcliffe, 2017; Zahavi, 2017) - as well as a discussion of how deflationary one should take the concept of pre-reflective self-awareness to be - e.g., whether it implies awareness of selfhood, or the intrinsically subjective aspect of phenomenal experience, or consciousness’s anonymous self-acquaintance - is beyond the scope of this paper (cf., Dainton, 2008; Gallagher, 2017, 2023; Guillot, 2017; Zahavi, 2017, 2020; Frank, 2022; Lang and Viertbauer, 2022). In any case, we have already argued that the pre-reflective self-awareness in flow is bodily but also suffused with a non-propositional, non-conceptual sense of know-how, control and worldly directedness (Zahavi, 2017, p. 196). In section 2.6 we will add to this picture, showing that the sense of self-as-subject in flow is marked with a (non-conceptualised) positive valence. As an intermediary conclusion, therefore, we hold that in flow the phenomenal self-as-subject is somewhat multifaceted and enhanced, yet, nevertheless, pre-reflective (cf., Legrand, 2007a,b).

“Thicker” phenomenological self-modelling can be found in complex systems with sufficient temporal depth, in the sense that such systems model regularly observed patterns in the environment, including the sensory outcomes of self-generated action, and can utilise this accumulated knowledge to contextualise and constrain real-time action selection (i.e., plan), as well as retrospect via long-term memory (Friston, 2018; Levin, 2019; Deane, 2021; Fields et al., 2023). This, in turn, permits counterfactual inference — the simulation of the sensory data an organism would observe if they were to enact some action policy in some given world — about optimal action sequence (policy) selection in the pursuit of minimising EFE (FitzGerald et al., 2014; Corcoran et al., 2020; Parr and Pezzulo, 2021; Vilas et al., 2022). As mentioned above, this has been described in terms of allostasis and is associated with certain phenomenological features of an ESM. These notably include reflective self-as-object-awareness enriched with temporal depth, such that, in frequent cases of planning, a self-as-object is experienced as having been projected forward and backwards in time. This is accompanied by a sense of epistemic agency, as I pre-reflectively experience myself driving this attentional time-travel (Metzinger, 2013a,b, 2015, 2017).

At the “thickest” level of phenomenological self-modelling is the conceptualisation of oneself as a *thing* that persists through time and is, thus, imbued with historicity. This is the quintessential form of the phenomenal self-as-object and can be called the conceptually-represented-self-as-object. Under Metzinger’s taxonomy, this form of self-awareness also belongs to an ESM.

Note that the two phenomenal features of the ESM we are analysing in this paper — what we call the temporally-extended-self-as-object and the conceptually-represented-self-as-object — are deeply connected. Firstly, as we have been stressing, both types of ESM involve some degree of reflective self-awareness. In planning, the self is experienced as an entity projected into an imagined future or recalled past. In moments of meta-conceptualisation, the self is experienced as a historicised object. Furthermore, the sense of being a conceptual self is an experience reserved for organisms endowed with a deep, temporal generative hierarchy, insofar as the very construction of the self as a historicised concept rests on the continuous thread of self-related aspects (preferences, dislikes, habits and so on) from the past into the future (Parfit, 1984), therefore implicating prospection and retrospection in the emergence of both the temporal and conceptual self-as-object (cf., Dennett, 1991; Seth, 2009; Damasio, 2012; Hohwy and Michael, 2017; Friston, 2018). In fact, counterfactual planning rests on the maintenance of this thread, since, in short, I choose what I do in the future based on what worked well for me in the past. These preferences are putatively encoded in the “higher levels” of the generative model, since, as described above, these levels track slower fluctuations in the external dynamics, constraining and contextualising the faster informational flows at the “lower levels” (Friston et al., 2010, 2017a; Clark, 2013, 2015; Pezzulo et al., 2018). In encoding slower trajectories, the higher levels inevitably encode statistical regularities which are isomorphic to the most deep-rooted and temporally invariant aspects of the organism’s being. Thus, the very predictions used to select policies stem from the more context-invariant information flows unfolding at the higher levels of the generative hierarchy. In other words, the very beliefs that the organism utilises to plan its behaviour are those which most define it at that moment, because they encode with a high probability the characteristic states which, in order to persist as the same organism, it must frequent (Hohwy and Michael, 2017).

Nevertheless, planning need not involve an explicit sense of the conceptually-represented-self-as-object; rather, in cases of planning where there is an intended sense of self, the cognitive system implicitly utilises the beliefs that *underwrite* the self-concept (except in cases of self-delusion; cf., Marchi and Newen, 2022) to select a policy in line with its preferences, projecting a non-propositional and non-historicised self-image into the past and future to assess the validity of possible policies. Conversely, a historicised conceptually-represented-self-as-object emerges from a post-hoc and higher-order propositional inference over these beliefs, engendering the hypothesis that there is a fixed *me*, which, notably, can exhibit and has exhibited acts of mental autonomy, such as planning (Metzinger, 2017; Fields et al., 2024). As mentioned above, such a case of self-conceptualisation is one in which Bayesian beliefs converge with (but do not collapse into) psychological beliefs. Of course, however, planning might involve the conceptually-represented-self-as-object, as in cases where action is taken in its service (e.g., when I decide (plan) to go to rehab because I no longer want to be an addict (i.e., I want to change my self-concept)). A more thorough discussion of the phenomenal interplay between the temporally-extended-self-as-object and the conceptually-represented-self-as-object is beyond the scope of this paper and ought to be pursued elsewhere.

Returning to the question of flow states, it is worth recognising that, according to active inference, the maintenance of the ESM – and, thus, its self-reflective aspects – is contingent on the correspondence of predicted and realised sensory data generated through action (Hohwy, 2007; Hohwy and Michael, 2017). This is because the alignment of prediction and reality affords the organism the possibility to infer itself as an effective, agentive cause of self-evidencing outcomes (cf., Frith, 2012). More precisely, it is this alignment which grants the organism – which can now take itself to be an agent – confidence in its own belief that it can endogenously bring about desired outcomes. Given this claim, it might seem paradoxical that individuals consistently report a loss of certain dimensions of self-awareness associated with the ESM in flow states. Indeed, the flow experience is induced when there is an appropriate balance between the perception one has of the challenges of the task and one’s relative competence, which must be maintained throughout the fulfilment of the task and thus requires that the predictions one makes about one’s behaviour leads to expected (sensory) outcomes (Nakamura and Csikszentmihalyi, 2009, 2014). What this suggests is that flow states and the ESM (including its self-reflective elements) are both rooted in the fulfilment of predictions about the sensorial consequences of action. Indeed, when there is a divergence between what the individual anticipates — as a result of their actions — and the subsequent outcome, self-reported experiences of flow are greatly reduced (Sidarus and Haggard, 2016; Vuorre and Metcalfe, 2016). Herein lies the puzzle: if the system’s confidence in its inferred ability to control sensorial outcomes underpins the emergence of the ESM, why do certain phenomenological markers associated with an ESM – i.e., the reflective temporal and conceptual sense of self – become lost in flow states, where evidence for that inference appears to be garnered?

The answer to this question lies in the fact that in flow states, what individuals reliably call a loss of self-consciousness just is the *temporary* loss of the phenomenal *self-reflective* contents of the ESM, what we have been calling the conceptually-represented-self-as-object and the temporally-extended-self-as-object, and that this loss is the result of the idiosyncratic environmental constraints flow places on the embodied cognitive system. These constraints do not undermine the developed ESM in the *long-term*; rather, they transitively hide its reflective aspects, whilst maintaining pre-reflective subjectivity expressed through the body. The principal question thus becomes why flow states inhibit reflective self-awareness and, in particular, what we have been calling the experience of the conceptually-represented-self-as-object and temporally-extended-self-as-object.

With respect to the former, there is simply no need (nor the capacity) to conduct the post-hoc inference that there is a fixed self when one is in flow.^22^ Indeed, to intend this conceptually-represented-self-as-object is synonymous with granting this (internal) object conscious attention. As has been explicated above, flow is so attentionally absorbing that the individual simply *cannot* reflect on itself as a concept *and* complete the at-hand task in flow at the same time. In terms of the latter, the processes that underwrite the temporally-extended-self-as-object are activated when there is a need for deep mental ‘time-travel’ – both retrospection and prospection – which is precluded in flow (Buckner and Carroll, 2007; Metzinger, 2008, 2013a; Schacter et al., 2008; Graziano and Webb, 2015; Friston, 2018; Deane, 2020). This form of temporal projection is particularly pivotal to planning. Flow is thus unique because, for reasons outlined in the section above, it prohibits such planning, meaning that the cognitive system is not enjoying the attentional, epistemic exploration of its internal representational space to inform (counterfactual) policy selection in a way which would normally yield the phenomenological sense of epistemic agency – i.e., the pre-reflective sense of myself driving introspective attention (Metzinger, 2013b, 2017; Wiese, 2019) – nor the temporally-extended-self-as-object, through which the intended ‘me’ has been projected (by itself) across counterfactual time and space. Indeed, flow inhibits planning not only by shrinking the horizon of counterfactual action selection, but also because the consequences of actions in flow are highly predictable, and, thus, flow does not involve the resolution of epistemic ambiguity, which invariably requires a degree of planning. Furthermore, the attentionally absorbing nature of flow states also prohibits planning of *extraneous* behaviour, effectively working in concert with the diminished planning horizon that the task engenders to prohibit autonoetic consciousness directed at either the upcoming demands of the task or extraneous future/past affairs.

In making this claim, we distinguish between what the organism is *able* to do and what it *does* do in the here and now. Indeed, although the very possibility of the ESM – and its reflective features – is putatively contingent on the temporal depth of the organism’s generative hierarchy – given that temporal depth prevents the predictive system being stuck in an eternal Now – its actual activation in real-time is the result of the organism’s utilisation of this depth, whether that be retrospective or prospective. The loss of the reflective aspects of the ESM in flow states is thus not related to the organism’s *general capacity* to mentally ‘time-travel’, but its reduced *real-time ability* to do so because of the specific precision weighting modulations engendered by the flow-inducing task context and the shallow planning horizon flow’s inherent volatility engenders (Buckner and Carroll, 2007; Schacter et al., 2008).

We further suggest that this representational planning in conscious humans will involve a degree of symbol manipulation, most often in the form of propositional statements – ‘if I do that, then this will happen’ (cf., D’Argembeau et al., 2011; Morin et al., 2011; Loevenbruck et al., 2018). Indeed, Morin et al. (2011) found that planning was the most self-reported function of inner speech, and D’Argembeau et al. (2011) found that near-future-pointing thoughts generally serve action and often take the form of inner speech. Consequently, since flow disrupts the propensity to plan, it also disrupts the generation of symbolic representations. Self-conceptualisation – ‘I am this thing’ – also rests on the use of such symbolic systems (Budwig, 2000; McLean et al., 2007). The inhibition of this capacity in flow thus further explains why flow experiences are fundamentally *non-propositional*.

However, crucially, flow does *not* eliminate all forms of self-awareness. Rather, it affords a *pragmatic, bodily subjectivity*: a form of non-reflective, bodily self-awareness imbued with sense of control, know-how and familiarity directed not inwards but onto the external dynamics of the world and the body, as well a positive affect marking the experience as autotelic (see section 2.6) (Dreyfus, 1990; Thompson, 2007; Legrand, 2007a,b; Christoff et al., 2011). Importantly, this means that pre-reflective bodily self-awareness in flow states is not confined to the experience of the “transparent body” (Legrand, 2007b). If this were to be the case, then flowing organisms could only be said to experience the world in a bodily way. In fact, we claim that in flow the body is experienced as pre-reflectively agentive. In other words, it is *also* experienced as a “performative body” (Gallagher, 2005; Legrand, 2007b). This experience is not absent in everyday life; however, it is powerfully forefronted in flow in a manner that distinguishes it from other, more quotidian activities. Furthermore, the sense of the “transparent body” is not lost in flow; rather, it is the bodily mode through which the world is experienced.^23^ These two aspects of pre-reflective bodily self-awareness are powerfully demonstrated by Sudnow’s (1993, p. 152) account of his jazz improvisation:

*I sing with my fingers, so to speak, and only so to speak, for there is a new ‘I’ that the speaking ‘I’ gestures toward with a pointing of the music that says: It is a singing body and this I (here, too, so to speak) sings.*

Note that this is *not* to say that the body cannot, and does not, take itself to be an intentional object in flow. As Legrand and Ravn (2009) show in the case of dancers, individuals can direct attention to states of their bodies without *reifying* them – that is, without alienating the body’s subjective, performative sense of agency. For these authors, this marks the difference between non-reifying perception and reifying scrutiny. For our purposes, such dancers are often in flow (Jaque et al., 2020; Łucznik et al., 2021), indicating that their experience – as well as those of many others, whether they be martial art practitioners or surfers – might involve the body’s subjective performativity as well as a perception of the body itself. Crucially, this awareness of the body occurs in a non-reifying (although intentional) manner, meaning that the body-as–reified-object proper (*Körper*) does not emerge in experience; nor, importantly, does a reflective self-model, which includes, but often goes beyond, the sense of body-as-object.^24^

### Flow states, habits and effortlessness

It is important to distinguish flow states from habits, given that they are both rooted in the execution of action policies without pronounced deliberation (Friston et al., 2016; Maisto et al., 2019). Before doing so, however, it is worth outlining the technical connection between the probability of action policies and their expected sensory outcomes. The expected pragmatic value of an action essentially uses the probability of ensuing outcomes to score that policy’s probability. Thus, in the relevant context — within which certain outcomes are inferred to have high pragmatic value — it will be the actions which yield those outcomes that will be selected, because those are the actions the system expects to enact in order to fulfil its expectations about the sensory data it will observe. Indeed, the goals of action are specified not in terms of latent or hidden states, but the preferred sensory outcomes following action, and the policies that are selected are those the organism subpersonally believes will lead to these preferred outcomes (Albarracin et al., 2021).

Conversely, under the active inference framework, habits are acquired by executing action policies and inferring what action was taken in a given context. This information can then be stored as a prior probability, or value, over policies associated with specific states — encoded in the E tensor in POMDP schema — whereby, when the organism infers itself to be in a given context, the prior value of a policy influences the selection of the policy itself to a lesser or greater degree, dependent on how many times it has been selected before (Maisto et al., 2019). This is because the total probability of a policy is determined by a combination of habitual priors (E) and EFE (G): cf., Equation 2 (Parvizi-Wayne and Severs, 2024).

Equation 2: The Contribution of Prior Values and EFE to Action Selection.

$Q\left(u\right)=\sigma \left(E-G\right)$

*Note that the σ notation refers to a normalised exponential* – i.e.*, softmax* – *function.*

This equation shows that a policy with a high posterior probability has a high prior habitual value E and a low EFE G, which is in part predicated on preferred sensory observations encoded by a C tensor or its parameters c (see Equation 1).

The crucial difference between flow states and habits lies in the fact that, according to the active inference framework, the selection of habitual schemes does not require deliberative inference, in the sense that they involve a simple stimulus–response pattern and do not involve state value representations (Friston et al., 2016; Miller et al., 2019). Technically, this is described in terms of a state-action policy, mapping from *states* to actions directly. This means that habitual policies do not involve counterfactual planning; namely, mapping from *beliefs about states* to actions. In certain situations, this can be advantageous as it allows the organism to act and minimise free energy more rapidly (Friston, 2009). However, despite also resulting in decreased counterfactual depth, flow states involve more goal-directed behaviour as well as the activation of full active inference at the level of state-based inference (see Figure 1). In other words, flow states are *partially deliberative,* insofar as the embodied cognitive system is still selecting actions that (implicitly) optimise beliefs about states and is driven to do so given the high precision weight those beliefs hold (Friston et al., 2015). This is mirrored by the co-activation of neural networks associated with cognitive control and goal-directedness during flow states (Huskey et al., 2018b, 2022).

That said, the process which *elicits* flow is not *entirely* deliberative. Rather, the relevant *context* (e.g., a concert hall full of people) acts as a cue for a habitual mental action,^25^ i.e., the modulation of precision weighting over beliefs about the expected sensory observations (C precision), beliefs about action dependent state transition (B precision) and the beliefs about likely sensory outcomes (A precision). Therefore, entering flow depends on a contextually cued form of habitual *mental* action. We thus distinguish the mental action performed at the level of mental state inference and physical action performed the level of perceptual state inference (cf., Figure 1), whereby the former driven by a contextual cue; the latter through greater precision weighting over C (induced by the mental action) which increases the influence of EFE – and its minimisation – in the selection of pragmatic, overt action policies.

Having disambiguated unconscious habitual policies from the policies deployed during a flow state, we can ask how these two might come to reflect each other over time. As mentioned above, the prior over policies (i.e., habits) is learned from the *post-hoc* inference of which policy was selected in a given context. Therefore, as the person’s training progresses, it is plausible to assume that their prior over policies will encode a high probability over precisely those policies that they then select on the basis of inferring the pragmatic value inherent in the flow-inducing task (having done so in previous sessions). The result is that the policy one selects in flow is close or identical to one’s prior over policies.

Furthermore, this computational characteristic of policy selection, namely the deviation from prior policy beliefs (E), has been related to the phenomenology of effort (Parr et al., 2023) and may represent the computational mechanism underpinning the effortlessness commonly associated with flow states (Csikszentmihalyi, 1990). More precisely, the account of Parr et al. (2023) proposes that effort can be mathematically formalised as the KL divergence between context-sensitive beliefs about how to act (calculated in terms of EFE; i.e., G) and context-insensitive priors over action (E). In terms of flow, what the organism would mentally do in a context-sensitive fashion and what they would do in a context-insensitive fashion is highly similar; that is, they would increase precision weight over the relevant parameters (A, B and C) of their generative model regardless of their habits, because of the pragmatic value present in the flow state. As such, the KL divergence between E and G with respect to the mental action of deploying precision weight is small, and, thus, at least with respect to the model offered by Parr et al. (2023), flow feels effortless.

### Losing and finding flow

To continue our account of flow states, it is worth recognising the boundary conditions that might help us determine whether an individual is in flow or not. In doing so, we can start by noting the phenomenological fact that flow is not always disrupted by the emergence of prediction error. In fact, *to a certain degree*, error that does (inevitably) ensue from the organism’s actions will be continuously “explained away” by swift motoric behaviour whilst the organism is in flow, i.e., by embodied *skill* (Clark, 2013; Bruineberg and Rietveld, 2014; Bruineberg et al., 2018; Hipólito et al., 2021). In the case of our violinist, prediction error caused by a sub-optimal bow angle will, under states of skilful flow, be resolved by motor action. Formally speaking, this produces sensory outcomes which better fit her predictive posterior.

This dynamism flexibility underpinning flow states is not trivial. Flow states do not involve a singular elimination of free energy and the ensuing cessation of action for the at-equilibrium organism. Rather, free-energy minimisation is a continuous demand on any self-evidencing entity. Thus, flow states necessarily entail peaks and troughs in prediction error within shallow perception-action loops. It is for this reason that we speak of a *repertoire* of action policies. No flow-inducing task performance involves only one course of action, and the power of strong procedural knowledge, encoded in motor pathways, lies in its flexibility and how it affords the organism the capacity to sequentially engage multiple action choices without invoking higher-order abstract thought. To account for these dynamic, protracted bouts of embodied skilfulness, we return to the notion of contextual cues and their role in triggering high precision weight over beliefs about preferred sensory outcomes and the way action unfolds over time. More precisely, we posit that, in flow, a sensory observation at time step *n* constitutes a contextual cue for precise beliefs about the action policy to unfold at *n + 1,* thereby attenuating precision weight over beliefs about the action policy at *n* as that time step comes to a close (see Hohwy et al., 2016; Parvizi-Wayne, 2024a for how this may relate to flow’s temporal phenomenology, as well as the specific empirical predictions different models of temporal passage make). In turn, the sensory observation made at *n*+1 becomes the contextual cue for the precision weighting dynamics governing action at *n*+2, and so on, affording a fractal or tree-like structure to extended bouts of skilled, flowing action and thereby imbuing those sequences with a degree of flexibility and sensitivity to environmental conditions.

Crucially, this is all achieved *implicitly*. This additional point explains the non-propositional nature of flow states, for if prediction error is not contextualised by such bodily dynamics, it would penetrate levels of the hierarchy associated with abstract, conceptual and linguistic thought (Dietrich, 2004). In this case, the flow state and its phenomenal condition breaks down. This might happen, for example, when the violinist plays a completely wrong note, rather than just a slightly suboptimal version of the correct note (Nakamura and Csikszentmihalyi, 2009, 2014). We consider this to be the moment in which the system, flooded with a rapid onset of uncertainty, turns in on itself, and asks itself what should be done in an environment that no longer seems to be offering exploitable, pragmatic affordances, but, rather, epistemic ambiguity – often about what should be done next – which needs to be resolved.^26^ Phenomenologically, in such cases a sense of self-as-object emerges (although the sense of self-as-subject is never lost; rather, it just seems to intend the self-as-object), as the phenomenal world transforms into an arena over which that self appears to plan, often in order to resolve such uncertainty (Dreyfus, 2014). In certain cases, this might even trigger meta-cognitive self-reflections – i.e., the system explicitly examines what *it* is (as is often the case in moments of embarrassment)^27^. We believe that, mechanistically, this is the result of a sudden increase in precision weight deployed on the higher levels of the generative hierarchy, as the organism, via planning, seeks information in order to plan its subsequent actions, with the ultimate goal of re-establishing flow (Friston et al., 2015).

In addition to such instances when the *whole* activity which had been engendering flow is interrupted, there are situations in which flow *per se* might be disrupted even if the activity, which had previously been the source of flow, continues. For example, Montero (2013, pp. 312–313), drawing upon her own experience as a ballet dancer, cites explicit, self-reflective thoughts she would have during dancing, such as “I am going to nail that coming balance.” This imposition of the sense of self-as-object involved in prospection emerges as a form of *preparation* for a particularly tricky balance, marking a break in the flow state even as the dance continues in an automatic, yet non-phenomenally-flowing, fashion. Thus, the loss of flow is not always observable externally; rather, it is recognised first and foremost by the conscious organism.

Notably, flow states become broken not only when task demands appear too extreme, but also when they are too simple. As mentioned above, for an organism to enter a flow state, its skillset must *match* the demands of the task (the so-called *balance hypothesis;* Csikszentmihalyi, 2003; Keller et al., 2011; Fong et al., 2014; Kennedy et al., 2014; Harmat et al., 2015; Baumann et al., 2016; Tozman et al., 2017). If, upon beginning a task, the highly adept organism senses that their capacities far exceed the demands of the task, boredom follows, but not flow (Csikszentmihalyi, 2002, pp. 155–157). The bored organism then turns to epistemic exploration, or, more precisely, novelty-seeking — technically, maximising information gain about model parameters — since there is little information to gain about the states of the world as they are (Sterling, 2012; Schwartenbeck et al., 2013; Gomez-Ramirez and Costa, 2017; Danckert, 2019; Maisto et al., 2019; Darling, 2023).

This indicates that reducing prediction error itself is not enough to enter flow. Rather, flow states require a certain degree of complexity for several reasons. Firstly, a sufficiently simple task — given the competencies of the organism — *does* permit deep temporal planning, since precisely weighted predictions over the outcomes of action and expected sensory data can be made further into a future which is not believed (explicitly or implicitly) to be volatile. This thus violates the essential conditions that yield flow’s phenomenology. Secondly, a simple task does not call for the near entirety of the organism’s attentional resources and thereby frees up precision weight deployment onto the likelihood distributions encoded within higher layers of the generative hierarchy, permitting planning and self-conceptualisation. Thirdly, recall that the environmental dynamics within which flow states unfold offer contextual cues to the organism which leads them to adopt precisely weighted beliefs over state transitions and the sensory data they expect to receive. It is this computational mechanism which drives the selection of pragmatic action over epistemic action in the pursuit of minimising EFE and this additional dimension which explains why the organism, literally *bored out of flow*, seeks out novelty: with the loss of challenge comes the loss of the contextual inference that drives precision weighting over the second-order beliefs that are critical to flow. As a result, the organism stops possessing the context-driven, high precision weight beliefs about the states it expects to occupy and observe, and it is these which drive the pragmatic action manifest in flow states. In other words, in line with previous accounts that posit boredom as guiding the organism’s switches between exploration and exploitation, sufficiently easy tasks provoke boredom because the transition away from pragmatic action towards novelty seeking is called for in the organism’s endless quest to minimise EFE (Gomez-Ramirez and Costa, 2017; Danckert, 2019; Darling, 2023).

Crucially, this situation of the bored organism differs from that in which the violinist plays a single erroneous note. Here, the sudden accumulation of free energy leads to the rapid attenuation of precision weight over the second-order beliefs about the outcomes of action and the expected sensory observations. In this context of uncertainty, the organism favours no singular goal state (because they do not know what goal state they prefer); in other words, the utilities of outcomes are same or similar, and, thus, policies cannot be distinguished in terms of the expected utility they might yield. In this case, policies are valuable if they maximise the entropy over outcome states, or the number of different outcomes the organism is likely to observe given a certain policy (Jaynes, 1957; Schwartenbeck et al., 2013; Parr and Friston, 2017). If the organism can resolve its uncertainty about the relevant states of the world, then precision weight over the second-order beliefs required for flow can be re-established as the organism prioritises the exploitation of affordances at hand to maximise expected utility. If, however, the contextual cue which leads to the flow-inducing precision weighting has now disappeared (e.g., the violin string has snapped), the bias towards the exploitation of pragmatic affordances is lost and flow is rendered unfeasible. This illustrates that the capacity to return to a flow state relies on the similarity of a given context to one which would usually induce flow.

### Flow is fun, but is it fun learning?

As we proceed in our survey of flow states, an open question remains: namely, whether flow states involve learning. For example, Vervaeke et al. (2018) suggest that flow involves implicit learning and, more specifically, “gaining intuition into real-world causal patterns, as opposed to correlational noise” (p. 22). More precisely, Vervaeke et al. (2018) conceptualise, building off the work of Hogarth (2001), “intuition as a result of implicit learning” (p. 21), whereby “the essence of intuitive responses is that they are reached with little apparent effort and typically without conscious awareness.” (Hogarth, 2001, p. 14 quoted in Vervaeke et al., 2018, p. 21). Similarly, Safron (2021a) proposes that flow involves the maximisation of *both* information gain and pragmatic value and occurs in the so-called “zone of proximal development” or Goldilocks zone, where tasks are just within the individual’s capacity (cf., Vygotsky, 1978; Kauffman and Clayton, 2006; Vasileva and Balyasnikova, 2019). Conversely, Dietrich (2004), although highlighting the engagement of the implicit system in flow, argues that flow is the execution of automatised behaviour. Vervaeke et al. (2018, p. 8) take this to mean that, because of Dietrich’s (2004) representationalist commitments, “his theory … entails no degree of qualitative growth or complexification of the system.”

The general idea that, as a result of being in flow, learning can occur, is, in and of itself, a perfectly reasonable claim. Indeed, it is likely that organisms learn more general patterns operating at longer time scales with respect to both their actions that they deploy in flow and their sensory consequences. For example, a skilled violinist might learn that they are prone to slight misalignments in their finger placement within a certain musical passage, and that these occurrences are always preceded by a mistimed horn-section.^28^ The question, however, is whether they are learning these patterns *in* flow or *after* flow. Crucially, insights like that of the violinist are often explicit and propositional; i.e., “I always make a mistake after the mistimed horn-section.” We, thus, suggest that any such explicit learning — based on this kind of insight — would be the result of post-flow inference, because flow precludes propositional thought *by its very nature*. However, as Vervaeke et al. (2018) stress, learning can also be *implicit* and not involve overt, propositional cognition (Reber, 1989; Seger, 1994; Cleeremans et al., 1998). In flow, therefore, it is plausible that learning could occur in terms of low-level sensorimotor contingencies hidden from the awareness of the flowing, embodied cognitive system (Friston et al., 2016). Again, the question of whether flow induces learning — and if so, how much — is open and empirically tractable, and we are keen to see this empirical work pursued elsewhere.

That said, it is worth recognising that the boundary between flow and not-flow is likely highly precarious, which means that, although skilful, adaptive action does licence its continuation, any bout of expert performance will involve a dynamic oscillation between flow and not-flow (Benson, 2003; Bergamin, 2017). This weakens the import of any claim that flow excludes learning, since, even if flow, when considered at its most atomistic level, involves only the execution of already-learnt patterns of action, any extended period of a flow-inducing activity will be marked by a constant fluctuation in and out of flow *proper* (cf., Wheeler and Cappuccio, 2010). It is plausible, therefore, that some of the learning, which flow facilitates, is achieved in these moments of no-flow within a broader context of flowing activity. Ultimately, this would mean that the question of whether flow involves learning — and its concomitant phenomenological features — might be, in part, determined by the lens of analysis one adopts.

Although the above example focuses on how much a skilled expert can learn in flow, it also sheds light on the education required before flow is even possible. Indeed, expertise is a prerequisite for flow, which means that novices must explicitly pursue epistemic, explorative behaviour in order to acquire the skill needed to tackle the complex, volatile situations in which flow experiences occur (Csikszentmihalyi, 1990; Nakamura and Csikszentmihalyi, 2009, 2014). This will involve tentatively and consciously sampling evidence for some action policy and how they map onto a subsequent state, before those actions become embedded into larger implicit, motoric schemes controlled by the basal ganglia (Mishkin et al., 1984), in a network that also involves the supplementary motor area, thalamus and hippocampus (Jenkins et al., 1994; Dietrich, 2004). Recalling our violinist once more, whilst playing her first concerto, she must *speak* herself through the experience, suffering the concomitant linguistic self-talk that accompanies exploratory behaviour. Only with time will the association between the relevant desired sensory outcomes (e.g., the right note) and the action that caused them be formed. At the same time, the habitual transition between a context and an action will be strengthened (Friston et al., 2016). More precisely, the context triggers a mental action which, in turn, sets high precision weight over expectations of how action will unfold over time and the sensory observations which will be made. These precision weighting dynamics govern the system’s prioritisation of exploitative behaviour, and their deployment are entailed directly by the action policies themselves (Schwartenbeck et al., 2013; Limanowski and Friston, 2018). In other words, learning to be in flow involves two concurrent developments: firstly, the association of action policies with desired outcomes; and, secondly, the connection between a given context and precision weight dynamics, which draws the organism towards the fulfilment of the task in a manner consonant with the phenomenology of flow.

For our violinist, this means that when she plays the same piece following a year’s practice, she can relinquish precision weight over her propositional knowledge, and place it wholly on the task dynamics at hand, thereby performing well without the impression of effort. This then grants her the attentional space for further exploratory behaviour, as epistemic action is stacked on top of already-acquired pragmatic skill as the individual pursues greater skill and fluency in a certain domain. Thus, becoming an expert is a protracted process, which requires the evolution of epistemic behaviour into pragmatic behaviour and the consequent return to exploratory behaviour at a higher degree of complexity without the loss of the more foundational procedural knowledge that has already been learnt (cf., Dreyfus and Dreyfus, 1986; Montero, 2010, 2013; Toner et al., 2015).

Nevertheless, we also suggest that opposing the learning associated with flow – whether that be *in* or *out* of flow – is a diachronous decline in the capacities and confidence of the organism. From the perspective of active inference, this can be explained by a hyper-prior the system possesses which expects that the world will change. This engenders a ‘forgetting’ of beliefs, including beliefs over action, whereby, without evidence to the contrary (i.e., practice), the precision weight of the beliefs implicated in flow will diminish over time (Moens and Zénon, 2019).

Crucially, we propose that this forgetting might be at the heart of the inherently rewarding, or autotelic, nature of flow states. This is because the flow state may provide a situation in which the person’s beliefs about their expected performance are revealed to be *overly* pessimistic. This pessimism is a natural result of the expected forgetting, described above, that occurs between sessions and leads the individual to accumulate uncertainty about their action-dependent transition beliefs as time passes, such that when they re-enter the flow context, the calculated EFE for a given policy is higher than it would have been previously when they had greater confidence in their actions. However, it is plausible that the embodied expertise inherent to flow means that this hyper-prior is overly pessimistic in this particular situation. In reality, picking up the violin again often feels “like riding a bike” and goes better than expected. As the person’s actions bring about expected outcomes in a manner that is better than predicted, the consequence, computationally under active inference, is an increase in their *model precision,* often denoted by a gamma parameter. This represents an uptick in the trust the person has in their own abilities and has been associated with positive valence (Hesp et al., 2021). The flow state might therefore be associated with a sense of joyfulness due to its ability to positively surprise our expectations of how well we should perform (Palomäki et al., 2021). This can be tied neatly to our idea that a pre-reflective, bodily self-awareness is made manifest in flow, since, as Solms and Panksepp (2012, p. 156) claim, “the phenomenal states of the body-as-subject are experienced affectively.”

This account is compatible with other theoretical accounts of valence which can be fruitfully applied to flow states. For example, building off the work of Van de Cruys (2017) and Kiverstein et al. (2019) suggest that “error dynamics” — the rate at which the embodied cognitive system is reducing prediction error — is at the heart of valence, such that “when an agent succeeds in reducing error at a faster than expected rate (or recognises the opportunity to do so) this feels good” (p. 2860), and *vice-versa* with respect to negative valence (see also Van de Cruys et al., 2020; Andersen et al., 2023; Kiverstein and Miller, 2023). As recent work by Fernández Velasco and Loev (2024) makes clear, Hesp et al. (2021)’s notion of “deeply felt affect” and that of “error dynamics” are convergent: with respect to flow, according to both accounts, the positive valence associated with flow states is rooted in the organism’s ability to reduce free energy at a better rate than they expected.

Affording that flow involves implicit learning of sensorimotor contingencies allows us to enrich this picture, since part of this better-than-expected free energy minimisation would be through learning and forming new, better predictions, and not only by making the world conform to preferences through action (Andersen et al., 2023). Thus, the positive valence of flow can be grounded not only in the return to a skill level *believed* to be lost, but also novel learning, with both factors likely at play simultaneously. This aligns flow with the broader notion of *play*, which has been proposed to be intrinsically rewarding because of the progress individuals are able to make in their learning (Oudeyer et al., 2007; Gottlieb et al., 2016; Oudeyer and Smith, 2016; Andersen et al., 2023). That said, as with play, any learning within flow would have to occur in the individual’s relative Goldilocks zone (Kidd et al., 2012). For reasons elucidated above, if the learning slope is too steep, flow will likely be broken and replaced by epistemic foraging. However, if a learning slope is too shallow, this is likely because the task is not sufficiently challenging and will consequently not yield the phenomenal state associated with flow.

## Conclusion

In this paper, we have posited a theory of flow states rooted in the active inference framework, which accounts for their unified phenomenology in terms of prediction and precision weight dynamics unfolding in the brain and body. Our central claim is that the neurocomputational basis of flow states is the allocation of high precision weight to second-order Bayesian beliefs about the consequences of action. In this context, when presented with a given task over which one has expertise, the exploitation of pragmatic affordances ensues. The specific phenomenology of flow, however, is contingent on, firstly, the deployment of high precision weight on the incoming sensory data and, thus, the wholesale deployment of attention on the task and the organism’s bodily engagement with it, and, secondly, the shallowness of the planning horizon it engenders. If these constraints are satisfied, flow ensues and brings with it its particular modulations to the self-awareness of the flowing organism. Although the literature surrounding flow states has recognised for decades that the flow experience involves changes to self-awareness relative to everyday life, this is, to the best of our knowledge, the first attempt to unpick *exactly* which aspects of the self-awareness are altered. We expect that qualitative work – especially via (micro) phenomenological interviews (Bevan, 2014; Petitmengin et al., 2019; Valenzuela-Moguillansky and Vásquez-Rosati, 2019) – will provide further evidence of our treatment; namely, that, in flow states, pre-reflective bodily self-awareness is retained – constituted by the experience of both the “transparent” and “performative” body – despite the elimination of what we have called the temporally-extended-self-as-object as well as the meta-cognitive conceptually-represented-self-as-object. Finally, we recognise that, in this paper, we have taken flow to be a bodily phenomenon. It would be interesting to consider if purely mental flow is possible and, if so, what phenomenology of self-awareness it might entail. In sum, there is a reason why flow has been considered an optimal state of being for free-energy minimising organisms like us. At its core, it is a sign that things are going well – in fact, better than expected – and that we are exercising our bodily skill in a complex environment that we need not be disengaged from, but which we can be coupled to.

## Data availability statement

The original contributions presented in the study are included in the article/supplementary material, further inquiries can be directed to the corresponding author.

## Author contributions

DP-W: Writing – original draft, Writing – review & editing. LS-S: Writing – review & editing. RP: Writing – review & editing. JL: Writing – review & editing. MT: Writing – review & editing. KF: Writing – review & editing.

## Footnotes

^1^We do not aim to suggest that the self-model theory of subjectivity, proposed by Metzinger (2004) and notably described as eliminativist with respect to the self (*cf.*, Metzinger, 2004, 2005a, p. 3), is the only relevant or plausible theory that can be brought to bear on the question of selfhood more generally, as well as in flow specifically. Furthermore, we recognise the ongoing theoretical tensions between different theories of the self, from which we borrow certain concepts and terminology (cf., Legrand, 2005; Zahavi, 2005; Metzinger, 2006; Legrand and Ruby, 2009). This paper is not an attempt to resolve these complex points of contention, nor provide an answer to the question of what the self *is*. Rather, we utilise Metzinger’s framework primarily because it provides a useful theoretical spectrum of self-awareness, from minimal phenomenal selfhood - which we associate with pre-reflective self-awareness – to epistemic phenomenal selfhood, which aids our analysis of self-awareness in flow states.

^2^We will continue to use the broader notion of the ESM in this paper (i) in order to distinguish between the sense of epistemic agency and diachronicity that is intrinsic to self-awareness in planning and (ii) given the fact that there can be a sense of epistemic pre-reflective self-awareness without a feeling of agency, as in mind-wandering, i.e., an ESM without an EAM (see text footnote 3) (Metzinger, 2018).

^3^It is important here to recognise the difference between planning via what Metzinger (2013a,b, 2015, 2017, 2018) calls mental (M-) autonomy – which will be the focus of this paper – and planning via mind-wandering. Metzinger (2015, p. 271) describes M-autonomy “the capacity to impose rules on one’s own mental behaviour, to explicitly select goals for mental action, the ability for rational guidance and, most importantly, for the intentional inhibition, suspension, or termination of an ongoing mental process.” Thus, whereas M-autonomy planning involves the intentional selection of goals for mental action in a manner that involves purposive mental time-travel (thereby rendering it constitutive of an EAM), planning via mind-wandering has been labelled “involuntary mental time travel” (Song and Wang, 2012, p. 5) and does not seem to involve a pre-reflective sense of epistemic agency associated with an EAM, despite maintaining the broader epistemic pre-reflective self-awareness associated with an ESM (cf., Dorsch, 2015; Christoff et al., 2016; Irving, 2016). That said, prospective mind-wandering has been shown to be directed at the planning of action in line with self-relevant future goals (cf., Berntsen and Jacobsen, 2008; Baird et al., 2011; Stawarczyk et al., 2011, 2013), suggesting that the notion of planning at its broadest level should accommodate both autonomous planning (associated with both an EAM and an EAM) and non-autonomous mind-wandering (associated with an ESM but not an EAM) (Metzinger, 2013a,b, 2015, 2017, 2018). A thorough treatment of the difference between autonomous planning and mind-wandering is beyond the scope of this paper, and, henceforth, we will use the term “planning” to refer to its purposive instantiation, recognising nevertheless that mind-wandering can involve planning and can be targeted at future action (as well as past retrospection) in such a way that a temporal self-concept emerges (i.e., ‘I have been projected into the past and/or future’), although without the pre-reflective sense of epistemic agency associated with autonomous attention (i.e., ‘I am the one projecting myself’) (cf., Metzinger, 2015, 2017, 2018). Similar claims might be made with respect to daydreaming (although see Lawson and Thompson, 2024). In any case, flow prohibits mind-wandering, daydreaming and autonomous planning as a result of the shallowness of the temporal horizon it engenders.

^4^We recognise that planning could be construed as an instrumental action. However, following Metzinger (2015, 2017), we will describe it as an epistemic capacity reserved for ESMs, insofar as it entails the emergence of a (pre-reflective) sense of self standing in an epistemic relation with the world, whether or not that involves a pre-reflective sense of epistemic *agency*.

^5^Second-order content-based approaches propose that episodic memories necessarily involve reflective self-awareness, as they are, in part, constituted by the sense of the self-as-object as *tied* to the historical event either temporally (Hoerl, 2001) or causally (Fernández, 2006). These researchers would thus refuse that self-awareness in episodic memory is purely pre-reflective.

^6^Recognising the confusion caused by the conflation of “for-me-ness,” “me-ness” and “mine-ness” in the literature on pre-reflective self-awareness, as highlighted by Guillot (2017), we will refer solely to the former in our analysis (cf., Zahavi, 2020; Lang and Viertbauer, 2022). Readers of this paper should thus interpret the terms “sense of self-as-subject” and “pre-reflective self-awareness” as referring to this phenomenal “for-me-ness,” which does not implicate an (overt) self-awareness. It is worth recognising that, for Guillot (2017, p. 35) “only in the “me-ness” and perhaps in the “mineness” readings is it [the self] (also) involved in the “accusative” position, as an object of phenomenal awareness.” Given results that suggest that the “flow experience shows a positive influence on consumers’ psychological ownership” (Yuan et al., 2021, p. 937), and following Guillot’s taxonomy, it could thus be proposed that flow states involve an (extremely thin) sense of self-as-object that is putatively imminent in the experience of “mineness.” However, it is unclear, as Guillot (2017) herself concedes, whether the experience of “mineness” genuinely does, by necessity, involve any reflective (i.e., objectifying) awareness of oneself as having an experience. Furthermore, “mineness” can be defined differently, and act instead as an adverbial term for how the objects of experience are presented to a non-objectified subject (cf., Rowlands, 2015, p. 117). Under this reading, as Zahavi (2020, p. 8) points out, “mineness” is no different than “for-me-ness.” Further analysis of this question is beyond the scope of this paper, however, we will, pace Zahavi (2020), implicitly understand “mineness” as the “mine-ly-ness” of experience, and not as an “introspectively discerned feature or property of my experiences” (p. 7) which implicates a self-as-object. As such, we shall continue to argue that flow inhibits the sense of self-as-object, but not the sense of self-as-subject, which involves an adverbal sense of possessive for-me-ness or “mineness.” Interestingly, Guillot (2017, p. 46) argues that “that the phenomenal character of a normal experience includes for-me-ness, me-ness, and mineness”; however, she does *not* explicitly demarcate “normal” and “abnormal” experiences, nor does she mention flow states, and, in any case, it is unclear that these three characters of experience *do* actually co-occur with any great regularity in everyday life (cf., Zahavi, 2020, p. 7).

^7^Note that the temporal sense of self-as-object generally implicated in planning is different from the epistemic sense of self yielded through introspective attention at an *object separate to me*. Whereas planning necessitates self-modelling at two levels: that of the planner (i.e., I (pre-reflectively) experience myself as directing attention) and the planned (i.e., I (reflectively) experience myself as having been projected across imaginary time), other manifestations of attentional agency only engender a pre-reflective experience of subjectively attending to something else (e.g., an imagined image of a loved one’s face) (Metzinger, 2015). In any case, this execution of introspective attention is prohibited too in flow.

^8^“The self is represented as something that stands in an epistemic relation to the world, in the relation of knowing, thinking, actively guiding attention, of just trying to understand what is going on.”

^9^Note that Pianzola et al. (2021) and Hohwy (2022) offer only brief accounts of flow from an active inference perspective.

^10^We thank Thomas Metzinger for reminding us of the ontological implications of the word “agent”.

^11^We recognise that modelling flow in terms of free energy minimisation through active inference involves a hybrid of discrete- and continuous-time variables, and so this paper will employ a combination of the languages of predictive coding employed for continuous state space models and discrete active inference modelling described by partially observable Markov decision process models (Friston et al., 2017a; Da Costa et al., 2023).

^12^Note that Figure 1 models an increase in the precision weighting of the likelihood mapping (ζ), which, as mentioned before, has been associated with attention in the active inference framework. Although high precision B and C matrices are necessary for the exploitation of pragmatic affordances, it is not the case, for reasons which will be outlined, that an extremely narrow attentional focus on the relevant task is needed in all such cases. Thus, it is worth stressing that the deployment of *extremely* high precision weight over ζ is unique to flow activities compared to other contextually cued behaviours, which, nevertheless, involve a degree of increased precision weighting over ζ for their fulfilment.

^13^Or what Heidegger might have called “circumspection,” i.e., the form of awareness which Dasein possesses when it is engaged with “tools-in-use” that have a “readiness-to-hand” as their form of Being in that moment (Heidegger, 2010).

^14^*It is this holistic background coping (disclosing) that makes possible appropriate dealings in particular circumstances (discovering). Only because, on entering the workshop, we are able to avoid chairs, locate and approach the workbench, pick out and grasp something as an instrument*, etc., *can we use a specific hammer to hit a specific nail, find the hammer too light or too heavy*, etc. (Dreyfus, 1990, p. 104).

^15^cf., Berliner (1994, p. 217): *No lead time separates conception from expression, and the gap between intention and realization disappears*.

^16^Note that Northoff et al. (2006)’s review compares “neural correlates during processing of stimuli related to the self with those of non-self-referential stimuli” (p. 440) and concludes that such neutral pathways form the DMN. This approach is called into question by Legrand and Ruby (2009), who suggest that the DMN is merely a correlate of self-*evaluation* and that the “the evaluative processes enabling identification, attribution, and reflection upon a subject are not different for self and others” (p. 270). To use their language, the DMN is not self-specifying; modulations in its activity “can be explained by the degree of involvement of inferences and memory recall” (Legrand and Ruby, 2009, p. 270). Crucially, the DMN would be consequently recruited not only for self-recognition and self-attribution, but also the recognition of and attribution to others. With respect to flow therefore, pace Legrand and Ruby (2009), the attenuation of the DMN in flow would be expected to weaken the processes of inference and evaluation, such as comparison, synthesis and induction (p. 268) that underlie self-attribution and self-identification - and are therefore implicated in planning and autobiographical self-reflection – without affecting the pre-reflective self-awareness intrinsic to experience itself. This accords with the claims made in this paper.

^17^Although in this section we have focussed on flow’s inhibition of temporal planning, it is worth remembering that the flow context inhibits all mental time-travel, a category which transcends pure planning. For example, the retrospection of one’s childhood (generally) involves mental time-travel and its dual self-related phenomenology – i.e., the emergence of a pre-reflective sense of epistemic agency and the reflective temporally-extended-self-as-object – without necessarily being geared towards current policy selection. That said, much planning involves retrospection and it has been argued elsewhere that the memory system evolved to aid future planning (cf., Klein et al., 2010). In this paper, we focus predominantly on planning because it is *directly affected* by the second of the two further constraints flow places on the embodied cognitive system – i.e., the prohibition of deep prospection given environmental volatility. Nevertheless, the constraint mentioned first – namely, the need for total attentional absorption in flow – disallows all forms of mental time-travel, including planning and retrospection.

^18^See Metzinger (2005a, p. 18) for the difference between *simulation* and *emulation*.

^19^The model of Legrand (2006, 2007a,b, Legrand and Ruby, 2009) does *not* ground the emergence of a self-specific perspective on the *matching* of efference and afference, unlike Frith et al. (2000) (and arguably the active inference models influenced by Frith’s *action monitoring* mechanism). Rather, the process Legrand has in mind is “more basic than Frith’s model because it requires only tracking of the afferent consequences of efferences, whether or not there is a matching of the respective informational contents” (Legrand and Ruby, 2009, p. 277). Although a thorough discussion of the differences between the two models is beyond the scope of this paper, it is interesting to consider whether the explanatory power of active inference – with respect to this section’s description of its application – is limited to the notion of self as contents attributed to the self which Legrand (2006, 2007a,b; Legrand and Ruby, 2009) argues are not self-specific. More broadly, it could be argued that the active inference models of selfhood constructed thus far can only account for the sense of self-as-object, not the sense of self-as-subject (cf., Woźniak, 2018; Nave et al., 2022).

^20^Although perhaps not the “thinnest” level; see Metzinger (2024) for a discussion of supposedly non-egoic, minimal phenomenal experiences.

^21^Although note that more phenomenologically-based, non active inference accounts (e.g., Hurley, 1998; O’Regan and Noë, 2001; Legrand, 2006, 2007a,b; Legrand and Ruby, 2009) do not give special significance to interoceptive content, given that, as put by Legrand and Ruby (2009, p. 272), such “self-related contents are not self-specific” (cf., Bermudez, 1998). Rather, they propose that the *processes* of sensorimotor integration – whether they be at an interoceptive, proprioceptive and exteroceptive level - are self-specifying and determine a *functional* distinction between self and non-self. Exteroception is given notable precedence in such accounts: e.g., “a perspective is defined as relating a perceiving subject and a perceived object. Our proposition is that this relation is made by the intertwining of the subject’s actions and their perceptual consequences in the world” (Legrand and Ruby, 2009, p. 276). Interoception is rarely mentioned (although see Christoff et al., 2011). Unfortunately, a thorough consideration of the differences between the active inference accounts that emphasise interoceptive free energy minimisation and earlier phenomenological accounts which focus on sensorimotor integration, as well as the validity of both approaches, are beyond the scope of this paper and should be examined elsewhere. Such commentaries should – to repeat the point made in footnote 19 – highlight the question of whether active inference models restrict the self to self-attributed contents which are not self-specific and, more broadly, the sense of self-as-object (cf., Legrand and Ruby, 2009; Hohwy and Michael, 2017; Woźniak, 2018). Additionally, they ought to highlight the differences between the MPS and the pre-reflective bodily sense of self which we have, for the sake of simplicity, aligned in this paper, despite each construct’s different theoretical commitments (cf., Legrand, 2005; Metzinger, 2006; Blanke and Metzinger, 2009; Zahavi, 2020; Gallagher, 2023). For example, Zahavi (2020, p. 13) argues that identifying the weak first-person perspective purely as a geometrical origin “*does not at all target the relevant subjective or experiential character of the first-person perspective. Not only would a robot presumably also possess the weak first-person perspective in question, although there is nothing it is like for it to undergo such presentations, but consider also something as self-involving and first-personal as emotions. Consider feelings of joy, anger, jealousy, despair or shame. It is somewhat hard to see how their first-personal character, their for-me-ness, could at all be explained or explicated with reference to the fact that the experiencing subject seems to have a determinate spatial location.*”

^22^Metzinger (2013a, p. 5) makes the additional suggestion that mind-wandering might be involved in “the constant creation and functional maintenance of the representation of transtemporal continuity, a fictional self.” A discussion of the lucid arguments he makes are beyond the scope of this paper but, given that mind-wandering is prohibited in flow, his claims are certainly compatible with our overarching proposal.

^23^We do not explicitly try to explain why flow forefronts the phenomenal experience of the “performative body”; rather, we focus on the attenuation of reflective aspects of self-awareness in flow and the maintenance of pre-reflective bodily self-awareness which is comprised of both a forefronted experience of the “performative body” and the experience of the “transparent body.” Legrand (2007b, p. 502) argues that that the forefronted experience of the “performative body” in dancers, who will often be in flow (Jaque et al., 2020; Łucznik et al., 2021), is rooted in the fact that, “in dance, goal and means collapse to some extent.” The validity of this claim with respect to all flowing organisms should be assessed elsewhere.

^24^This puzzle of bodily self-awareness raised by Legrand and Ravn (2009) raises an interesting auxiliary question: can the self (intentionally) perceive its subjectivity without taking itself as a reified object? (cf., Zahavi, 2005).

^25^Note that the precision over the prior probability (*E*^(2)^ in Figure 1) of this mental action (*u*^(2)^_1_ in Figure 1) will be high so that the mental action is driven by this habitual term.

^26^This is akin to what Heidegger describes as a “breakdown” case in which worldly concerns can present as “un-ready-to-hand.”

^27^We recognise here the potential contention that other forms of reflective self-awareness might be at play in such contexts. For example, the anxious violinist might think to themselves: "what have I done?!", a cognitive act which arguably does not strictly invoke a temporally-extended-self-as-object nor a conceptually-represented-self-as-object, as we have defined these constructs. However, our aim is not to delineate just how many types of reflective self-awareness exist, and our focus on two particular types should not be taken to exclude others which future studies might explore. Nevertheless, due to the unique constraints of the flow environment, whichever other forms of reflective self-awareness that one might imagine are inhibited too. For example, "what have I done?!" or "what will the audience think of me?!" might be said to elicit a particular category of self-reflection. Even if this is the case, that phenomenon emerges in contexts of uncertainty, of which flow states are not one.

^28^We thank Jakob Hohwy for this example.
