# Erratum: Forgetting ourselves in flow: an active inference account of flow states and how we experience ourselves within them

**DOI:** 10.3389/fpsyg.2024.1456438

**Published:** 2024-07-10

**Authors:** Frontiers Production Office

**Affiliations:** Frontiers Media SA, Lausanne, Switzerland

**Keywords:** active inference, embodied cognition, phenomenology, consciousness, self-awareness, predictive processing

Due to a production error, the conflict of interest statement was erroneously omitted. The statement reads as follows:

KF and RP were employed by the VERSES AI Research Lab.

The remaining authors declare that the research was conducted in the absence of any commercial or financial relationship that could be construed as a potential conflict of interest. The author(s) declare that they were members of the editorial board of Frontiers, at the time of submission. This had no impact on the peer review process or the final decision.

The publisher apologizes for this mistake. The original article has been updated.

